# Targeting the spliceosome through RBM39 degradation results in exceptional responses in high-risk neuroblastoma models

**DOI:** 10.1126/sciadv.abj5405

**Published:** 2021-11-17

**Authors:** Shivendra Singh, Waise Quarni, Maria Goralski, Shibiao Wan, Hongjian Jin, Lee-Ann Van de Velde, Jie Fang, Qiong Wu, Ahmed Abu-Zaid, Tingting Wang, Ravi Singh, David Craft, Yiping Fan, Thomas Confer, Melissa Johnson, Walter J. Akers, Ruoning Wang, Peter J. Murray, Paul G. Thomas, Deepak Nijhawan, Andrew M. Davidoff, Jun Yang

**Affiliations:** 1 Department of Surgery, St. Jude Children’s Research Hospital, 262 Danny Thomas Place, Memphis, TN 38105, USA.; 2 Department of Internal Medicine, Program in Molecular Medicine, University of Texas Southwestern Medical Center, 5323 Harry Hines Blvd. K3.124, Dallas, TX 75390, USA.; 3 Center for Applied Bioinformatics, St. Jude Children’s Research Hospital, 262 Danny Thomas Place, Memphis, TN 38105, USA.; 4 Department of Immunology, St. Jude Children’s Research Hospital, 262 Danny Thomas Place, Memphis, TN 38105, USA.; 5 Center for Childhood Cancer and Blood Disease, Abigail Wexner Research Institute, Nationwide Children’s Hospital, 700 Children’s Drive, Columbus, OH 43205, USA.; 6 Division of Radiation Biophysics, Department of Radiation Oncology, Massachusetts General Hospital and Harvard Medical School, Boston, MA 02114, USA.; 7 Center for In Vivo Imaging and Therapeutics, St. Jude Children’s Research Hospital, 262 Danny Thomas Place, Memphis, TN 38105, USA.; 8 Max Planck Institute of Biochemistry, Am Klopferspitz 18, 82152 Martinsried, Germany.

## Abstract

Aberrant alternative pre-mRNA splicing plays a critical role in MYC-driven cancers and therefore may represent a therapeutic vulnerability. Here, we show that neuroblastoma, a MYC-driven cancer characterized by splicing dysregulation and spliceosomal dependency, requires the splicing factor RBM39 for survival. Indisulam, a “molecular glue” that selectively recruits RBM39 to the CRL4-DCAF15 E3 ubiquitin ligase for proteasomal degradation, is highly efficacious against neuroblastoma, leading to significant responses in multiple high-risk disease models, without overt toxicity. Genetic depletion or indisulam-mediated degradation of RBM39 induces significant genome-wide splicing anomalies and cell death. Mechanistically, the dependency on RBM39 and high-level expression of DCAF15 determine the exquisite sensitivity of neuroblastoma to indisulam. Our data indicate that targeting the dysregulated spliceosome by precisely inhibiting RBM39, a vulnerability in neuroblastoma, is a valid therapeutic strategy.

## Teaser

Degradation of RBM39 by indisulam leads to exceptional response in high-risk neuroblastoma.

## INTRODUCTION

High-risk neuroblastoma has a poor prognosis and leads to a disproportionate number of cancer-related deaths in children, despite the use of increasingly intensive, multimodal therapies (*1*). Survivors of high-risk disease have a significant risk of developing long-term complications from cytotoxic chemotherapy and radiotherapy (*2*, *3*). Safer, more effective therapies are needed for high-risk neuroblastoma patients. However, the low number of targetable recurrent mutations in neuroblastoma remains an obstacle to the development of targeted therapies with high therapeutic indices.

MYC is one of the nuclear factors most often associated with dysregulated transcription, as genetic abnormalities of the *MYC* family of oncogenes (*C-MYC*, *MYCN*, and *MYCL1*) are among the most common alterations in human cancer (*4*, *5*). Neuroblastoma, a malignancy of arrested development of the peripheral nervous system (*1*, *6*), is a *MYC*-driven cancer (*7*–*9*). Half of high-risk neuroblastomas harbor *MYCN* amplification, and the other half overexpress *C-MYC* (*10*). Without a strategy to target the MYC protein directly, investigation has focused on identifying the pathways that MYC requires to drive cancer growth. To that end, pre-mRNA splicing has emerged as a potential vulnerability in MYC-driven cancers (*11*–*18*), including neuroblastoma (*19*). Splicing abnormalities are present in both human neuroblastomas and a mouse model of MYCN-driven neuroblastoma (*19*–*22*). The dependence of MYC-driven cancers on components of the spliceosome and the presence of splicing abnormalities in MYC-driven neuroblastoma raise the possibility that splicing factors could be effective targets in neuroblastoma.

The aryl sulfonamide drug indisulam has been shown to act as a “molecular glue” that bridges the U2AF-related splicing factor RBM39 to the CRL4-DCAF15 E3 ubiquitin ligase for proteasomal degradation of RBM39 (*23*–*28*). Recruitment of RBM39 to CRL4-DCAF15 causes RBM39 ubiquitination followed by proteasomal degradation, leading to splicing abnormalities within treated cells. The amount of RBM39 degradation is proportional to the expression of *DCAF15*, and therefore, the sensitivity of cells to indisulam is expected to depend on both RBM39 dependence and *DCAF15* expression. Here, we demonstrate that neuroblastoma is both dependent on RBM39 and expresses relatively high levels of *DCAF15*, providing a mechanistic rationale for indisulam treatment. Accordingly, when compared to other lineages, cultured neuroblastoma cells are significantly more sensitive to indisulam. Moreover, indisulam-mediated RBM39 degradation leads to complete tumor regression in multiple, independent mouse models of high-risk neuroblastoma. These observations provide a scientific rationale for treating high-risk neuroblastoma with small-molecule RBM39 degraders like indisulam.

## RESULTS

### High-risk neuroblastoma is characterized by heightened expression of RNA splicing genes and spliceosomal dependency

**Fig. 1.**
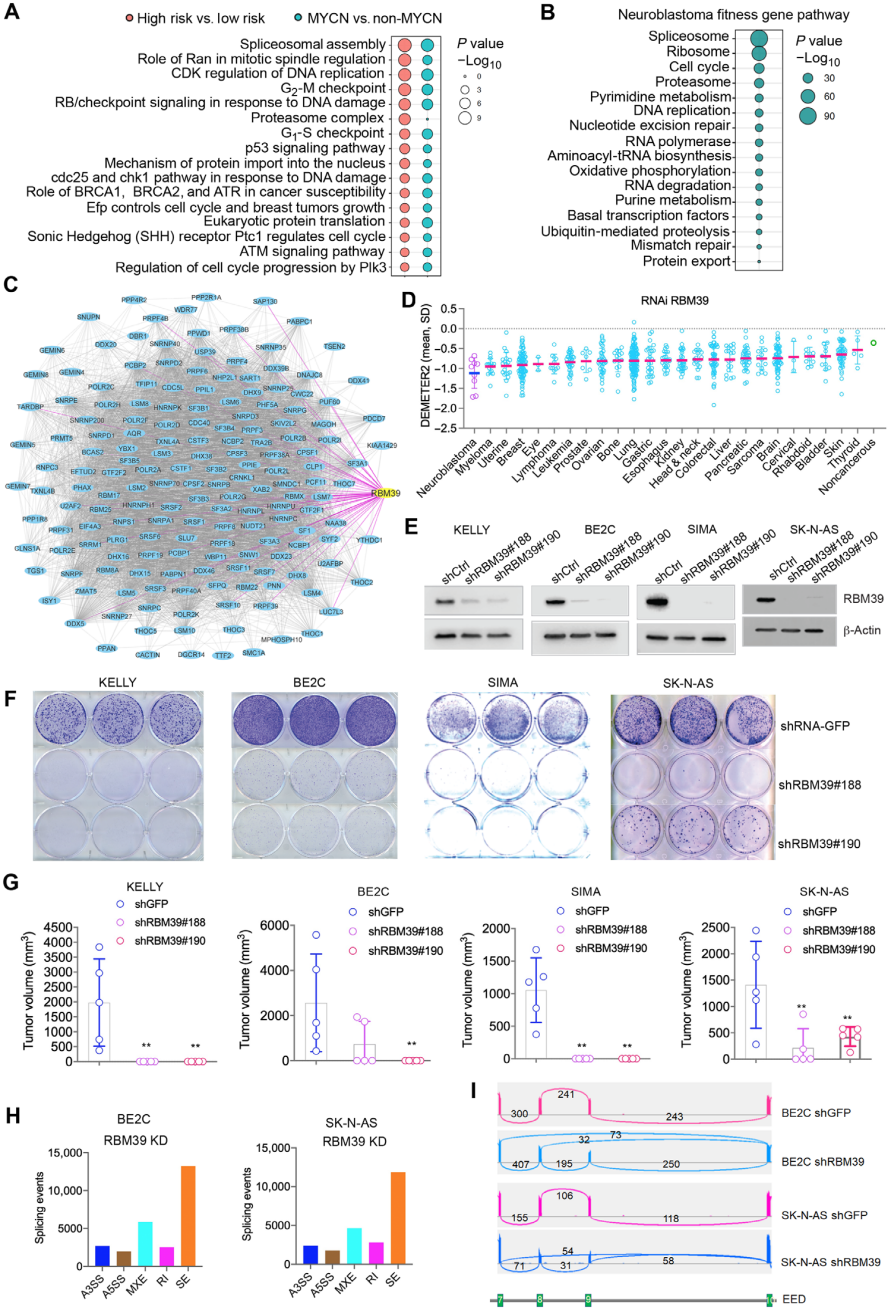
The splicing factor RBM39 is required for neuroblastoma cell survival. (**A**) Bubble plot showing the dysregulated signaling pathways with *P* values in −log_10_. (**B**) Bubble plot showing the dysregulated signaling pathways with *P* values in −log_10_. (**C**) RNA splicing network node from essential fitness gene network, in which RBM39 is highlighted in yellow. (**D**) DEMETER2 score (mean ± SD) of an RNAi screen across 25 cancer lineages. Neuroblastoma versus others, *P* = 0.0023. (**E**) Western blot to validate the RBM39 knockdown after lentiviral transduction of shRNA control [green fluorescent protein (GFP)] and two different *RBM39* shRNAs (#188 and #190) for 72 hours. (**F**) Colony formation for neuroblastoma cells transduced with lentiviral shRNA control and two different *RBM39* shRNAs followed by puromycin selection for 7 days. Cells stained with crystal violet for visualization. (**G**) Tumor volume comparison between shRNA control and *RBM39* knockdown by two different shRNAs in four tumor models. *P* values calculated using unpaired Student’s *t* test. (**H**) RNA splicing analyses for different events altered by *RBM39* knockdown in BE2C and SK-N-AS cells. The *y* axis indicates numbers for each splicing event induced by *RBM39* knockdown in comparison with shRNA control. A3SS, alternative 3′ splicing; A5SS, alternative 5′ splicing; MXE, mutually exclusive exon; RI, intron retention; SE, skipped exon. (**I**) Sashimi plot using IGV program shows the splicing changes of *EED* gene in BE2C and SK-N-AS cells after *RBM39* knockdown, which indicates the missplicing among exons 7 to 10. The numbers indicate the read counts of exon-intron junctions of RNA-seq.

To determine cellular pathways in high-risk neuroblastoma (generally, tumors with *MYCN* amplification or those that have metastasized in children >18 months of age at diagnosis), we analyzed the 3000 genes most differentially expressed between low- and high-risk neuroblastomas among 498 primary neuroblastoma samples (*29*). An analysis of pathways associated with these genes revealed “spliceosome assembly” as the most significantly enriched pathway in high-risk neuroblastoma (Fig. 1A and table S1), followed by DNA replication and cell cycle signatures, supporting the hypothesis that proteins important for splicing may be valid therapeutic targets for high-risk disease. Using a similar approach, we found that spliceosome assembly was the most enriched pathway in neuroblastomas with *MYCN* amplification (Fig. 1A). In line with this hypothesis, 1585 genes essential for neuroblastoma cell survival (*n* = 7, dependency score > 40%; table S2) were identified in a genome-wide CRISPR-Cas9 library screen by the Sanger institute (*24*). Pathway analysis revealed that these genes are enriched in regulation of RNA spliceosome components (Fig. 1B), which formed an interaction network (Fig. 1C). RBM39 is notable among these splicing factors because it can be degraded with small molecules. Within the subnetwork, RBM39 interacted with 79 splicing factors (Fig. 1C and fig. S1A; confidence score threshold = 0.4), which are enriched with MYC targets, PRMT5 targets, and tumor invasiveness (fig. S1B). To investigate whether RBM39 itself is a MYC target, we assessed the epigenetic landscape of *RBM39* genomic locus in a primary neuroblastoma with *MYCN* amplification, which showed MYCN binding at the promoter region of *RBM39*, in line with the binding of BRD4, CTCF, RNA polymerase II, and enrichment of active transcriptional marks (H3K27Ac, H3K9-14Ac, H3K4me3, H3K4me2, H3K4me1, and H3K36me3) (fig. S1C). We further overexpressed MYCN in SK-N-SH cells and performed real-time polymerase chain reaction (PCR) and Western blot, which showed that RBM39 was induced at mRNA and protein levels by MYCN (fig. S1, D and E). These data suggest that RBM39 is a direct target of MYC.

### RBM39 is required for neuroblastoma cell survival and tumor growth

DEMETER2 is a model that scores gene dependencies in 712 cancer cell lines that have been evaluated in three different large-scale pooled RNA interference (RNAi) screens (*30*), providing an additional opportunity to assess the dependency of neuroblastoma on RBM39. Although RBM39 is essential to most cancer cell lines, neuroblastoma was the lineage most significantly sensitive to RBM39 RNAi compared to all other lineages (*P* = 0.0023) (Fig. 1D and table S3). Among the splicing factors directly interacting with RBM39 (fig. S1A), genetic knockdown of many of them led to greater inhibition of neuroblastoma than other cancer lineages (fig. S2), supporting the hypothesis that neuroblastoma is relatively more dependent on splicing factors related to RBM39. We further validated these findings by stable expression of two different short hairpin RNAs (shRNAs) designed to target *RBM39* in four different neuroblastoma models, all of which are MYC-driven as a result of either *MYCN* amplification (BE2C, KELLY, and SIMA) or C-MYC overexpression (SK-N-AS) (Fig. 1E). *RBM39* depletion led to significantly reduced neuroblastoma cell colony formation in vitro (Fig. 1F) and tumor growth in vivo (Fig. 1G). An analysis of the transcriptome from BE2C and SK-N-AS neuroblastoma cells with *RBM39* depletion demonstrated alterations in pre-mRNA splicing (particularly exon skipping) in comparison to vector controls (Fig. 1H, fig. S3, A to H, and tables S4 and S5). For example, the *EED* gene that encodes a critical component of polycomb-repressive complex 2 that plays a critical step in neuroblastoma growth (*31*, *32*) showed exon 8 and 9 skipping after *RBM39* depletion (Fig. 1I). By comparing with the cancer genes from COSMIC (Catalogue Of Somatic Mutations In Cancer) database, we found that a number of misspliced genes by *RBM39* knockdown were overlapped with COSMIC cancer genes (table S6), which are involved in a variety of key cancer pathways such as cell cycle (*CDK4* and *CCND3*), apoptosis (*BAX* and *BIRC6*), DNA repair (*ATM*, *ATR*, and *MLH1*), tyrosine kinase receptor–mediated signal transduction (*NTRK1*, *FGFR1*, *FGFR4*, *RET*, and *MAP2K2*), epigenic regulation (*EED*, *EZH2*, *DNMT3A*, *ARID1B*, and *KDM6A*), and mitochondrial metabolism (*SDHC* and *SDHAF2*), suggesting that RBM39 is a critical factor in the oncogenesis of neuroblastoma. Real-time PCR for specific exons spliced by *RBM39* knockdown validated the RNA sequencing (RNA-seq) results (fig. S3, I to L). Western blot showed that the expression of these targets was reduced by *RBM39* depletion (fig. S3, M and N). The splicing changes we observed for cells expressing each individual shRNA in both cell lines showed a large number of common splicing events and significant correlation with each other, as indicated by the Spearman correlation and Venn diagram analyses (fig. S4), suggesting that the observed alterations were specific to RBM39 suppression. Gene set enrichment analysis (GSEA) for the differentially expressed genes showed that RBM39 depletion significantly disrupts pathways that regulate RNA and protein homeostasis (fig. S5, A and B, and table S7). Collectively, these data indicate that neuroblastoma has an inordinate dependency on RBM39-mediated RNA splicing, which may represent a vulnerability in MYC-driven high-risk neuroblastoma (Table 1).

**Table 1.** Information for cell lines. CHLA20 has MYC amplification. ATCC, American Type Culture Collection; BM, bone marrow; Dx; diagnosis; DSMZ, German Collection of Microorganisms and Cell Cultures; ECACC, European Collection of Authenticated Cell Cultures; MNA, MYCN amplification; NB, neuroblastoma; PD, progressive disease; RA, right adrenal mass; N/A, not available.

**Cell lines/PDX**	**Class**	**Stage (1–4)**	**Age (months)**	**Gender**	**Origin**	**Phase of** **therapy**	**MYCN status**	**Source**
SJNB11	PDX	4	22	M	RA	PD	MNA	St. Jude
SJNB14	PDX	4	24	M	RA	PD	MNA	St. Jude
SJNB19	PDX	4	84	M	RA	PD	Non-MNA	St. Jude
NGP	NB cell line	N/A	30	M	Lung	PD	MNA	DSMZ, ACC676
SKNDZ	NB cell line	N/A	24	F	BM	N/A	MNA	ATCC, CRL2149
KELLY	NB cell line	N/A	12	N/A	N/A	N/A	MNA	ECACC, 92110411
SIMA	NB cell line	3	20	M	Kidney	PD	MNA	DSMZ, ACC164
BE2C	NB cell line	4	24	M	BM	PD	MNA	ATCC, CRL2268
SK-N-AS	NB cell line	4	96	F	BM	PD	Non-MNA	ATCC, CRL2137
SK-N-SH	NB cell line	4	48	F	BM	PD	Non-MNA	ATCC, HTB-11
CHP212	NB cell line	N/A	20	M	Kidney	Dx	Non-MNA	ATCC, CRL2273
CHLA20	NB cell line	4	18	F	Kidney	PD	Non-MNA	COG
SK-N-FI	NB cell line	N/A	132	M	BM	PD	Non-MNA	ATCC, CRL2142
HS68	Fibroblast							ATCC, CRL1635
BJ	Fibroblast							ATCC, CRL2252

### The RBM39 degrader indisulam is selective and highly active against neuroblastoma in vitro and in vivo

**Fig. 2.**
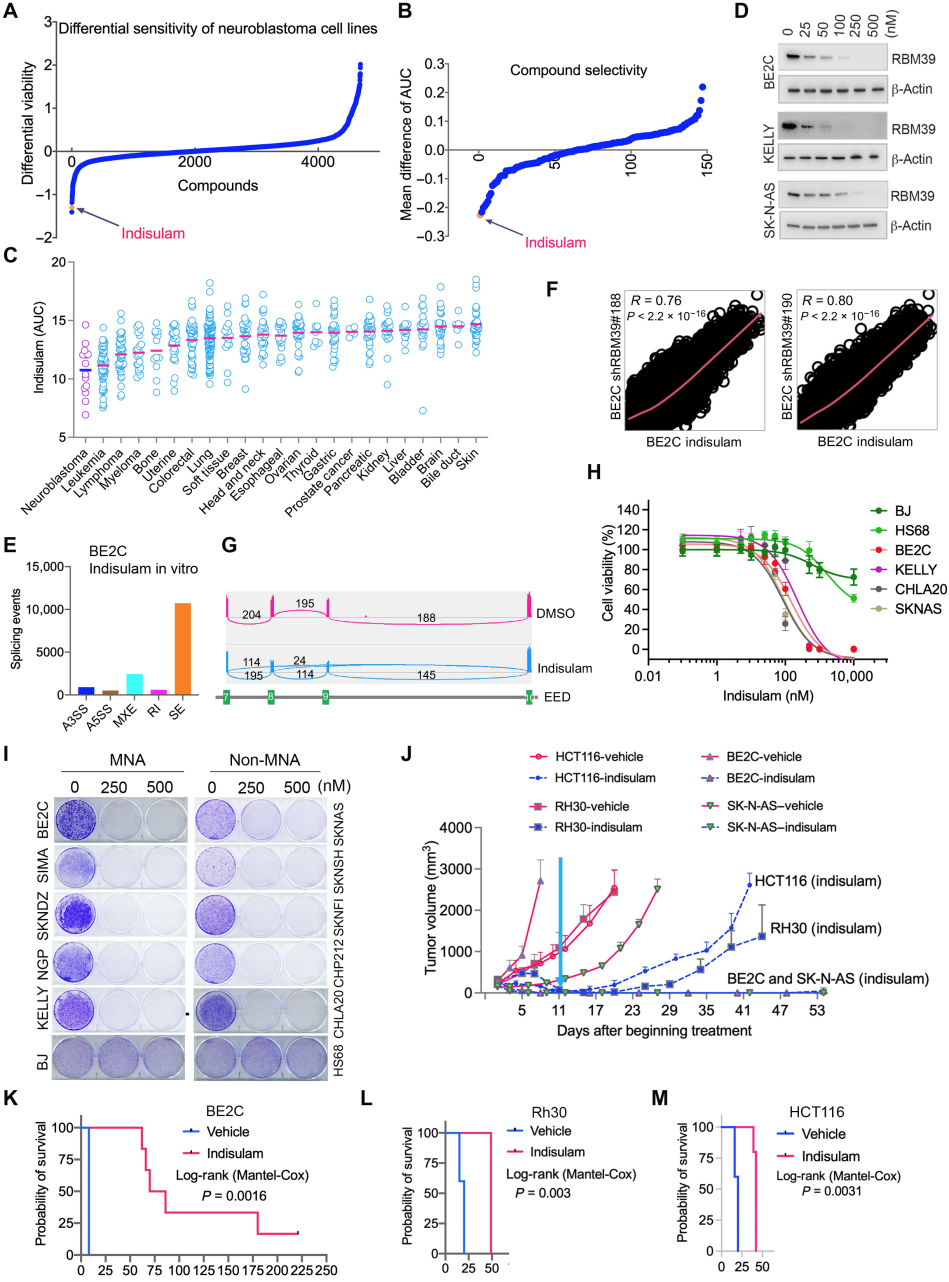
The RBM39 degrader indisulam is selectively and highly active against neuroblastoma in vitro and in vivo. (**A**) Differential mean toxicity plot for neuroblastoma cell lines (*n* = 5) treated with single doses of 4686 compounds by PRISM. (**B**) AUC screen by PRISM showing indisulam selectivity against neuroblastoma cell lines (*n* = 4). (**C**) AUC of indisulam against neuroblastoma cell lines (*n* = 14) and other cancer lineages by CTRP (*60*). (**D**) Neuroblastoma cells were treated with indicated concentrations of indisulam for 72 hours, followed by Western blot analysis of RBM39. (**E**) Analysis of splicing events in BE2C cells altered by 250 nM indisulam treatment for 72 hours. (**F**) Sashimi plot shows *EED* missplicing in exons 7 to 10 in BE2C cells by indisulam. The numbers indicate the read counts of exon-intron junctions of RNA-seq. (**G**) Genome-wide pairwise correlation analysis of altered splicing events induced by indisulam and *RBM39* knockdown. (**H**) Dose-response curve shows the differential effect of indisulam on neuroblastoma cells and normal human cell BJ and HS6. (**I**) Colony formation assay for cell lines treated with indisulam for 7 days. (**J**) Mean tumor volume for BE2C (vehicle = 5; indisulam = 6), SK-N-AS (vehicle = 7; indisulam = 8), HCT116 (vehicle = 5; indisulam = 5), and Rh30 (vehicle = 5; indisulam = 5) xenografts treated with indisulam (25 mg/kg), 5 days on and 2 days off for two cycles. Cyan arrow indicates when therapy discontinued. (**K** to **M**) Kaplan-Meier survival curve for BE2C (K), Rh30 (L), and HCT116 (M) xenografts.

Indisulam and other related aryl sulfonamides represent a class of compounds known as “molecular glues” because of their ability to recruit the splicing factor RBM39 to the E3 ubiquitin ligase CRL4-DCAF15 for proteasomal degradation (*23*, *28*). We reasoned that indisulam might be an effective agent for targeting the splicing program in MYC-driven neuroblastoma by degrading RBM39. Profiling Relative Inhibition Simultaneously in Mixtures (PRISM) and the Cancer Therapeutic Response Portal (CTRP) are two independent large-scale datasets that tested the impact of many drugs, including indisulam, on hundreds of different cell lines, providing a resource to identify neuroblastoma-selective compounds. Primary PRISM evaluated the impact of 4686 different small molecules on the proliferation of up to 560 human cancer cell lines. A secondary analysis measured the sensitivity of 148 compounds using a dose titration and quantified sensitivity as assessed by determining the area under the dose-response curve (AUC). Ranking the compounds in order of selectivity for neuroblastoma revealed indisulam to be third out of 4686 and first out of 148 compounds in the primary and secondary analyses, respectively (Fig. 2, A and B; fig. S6, A and B; and tables S8 and S9). In the CTRP analysis of 709 cell lines, the indisulam AUC for neuroblastoma showed it to be significantly smaller (more sensitive) than all other cell lineages (Fig. 2C, fig. S6C, and table S10). Consistent with these predictions, we found that indisulam induced dose-dependent reduction of RBM39 (Fig. 2D) and widespread splicing anomalies. The splicing anomalies were due to RBM39 degradation, because genome-wide pairwise comparisons between cells expressing *RBM39* shRNA and those treated with indisulam showed a significant correlation (Fig. 2, E to G), in line with the common splicing events by Venn diagram analysis (fig. S7). A PrestoBlue assay for viability showed that neuroblastoma cell lines were more sensitive than select normal human cell lines BJ and HS68 (Fig. 2H). In addition, indisulam reduced colony formation in 10 different neuroblastoma cell lines (Fig. 2I). Flow cytometry analysis showed that indisulam induces G_2_-M phase arrest and cell death, as indicated by an increase in sub-G_1_ phase cells (fig. S8). Pathway analysis of the genes with altered splicing by indisulam and RBM39 knockdown revealed that loss of RBM39 affected pathways involved in G_2_-M phase cell cycle, gene transcription, DNA repair, chromatin-modifying enzymes, metabolic pathways, and cancer genes (fig. S9). Real-time PCR and Western blot validated that the splicing events induced by indisulam were consistent with those induced by RBM39 knockdown (fig. S10, A to N).

We then tested the in vivo efficacy and selectivity of indisulam in various xenograft models, including neuroblastoma (BE2C and SK-N-AS), rhabdomyosarcoma (Rh30), and colorectal cancer (HCT116). Mice harboring tumors of approximately 200 mm^3^ were dosed intravenously with 25 mg/kg for two 1-week cycles (5 days on and 2 days off). Although all tumors in all four models underwent an initial complete response following treatment, the time to relapse, following discontinuation of therapy, in neuroblastoma was substantially longer in comparison to the Rh30 and HCT116 models (Fig. 2, J to M). Together, these data suggest that neuroblastoma is more sensitive to RBM39 degrader indisulam in comparison with other cancer lineages.

### Indisulam efficacy against neuroblastoma is specifically through targeting RBM39

**Fig. 3.**
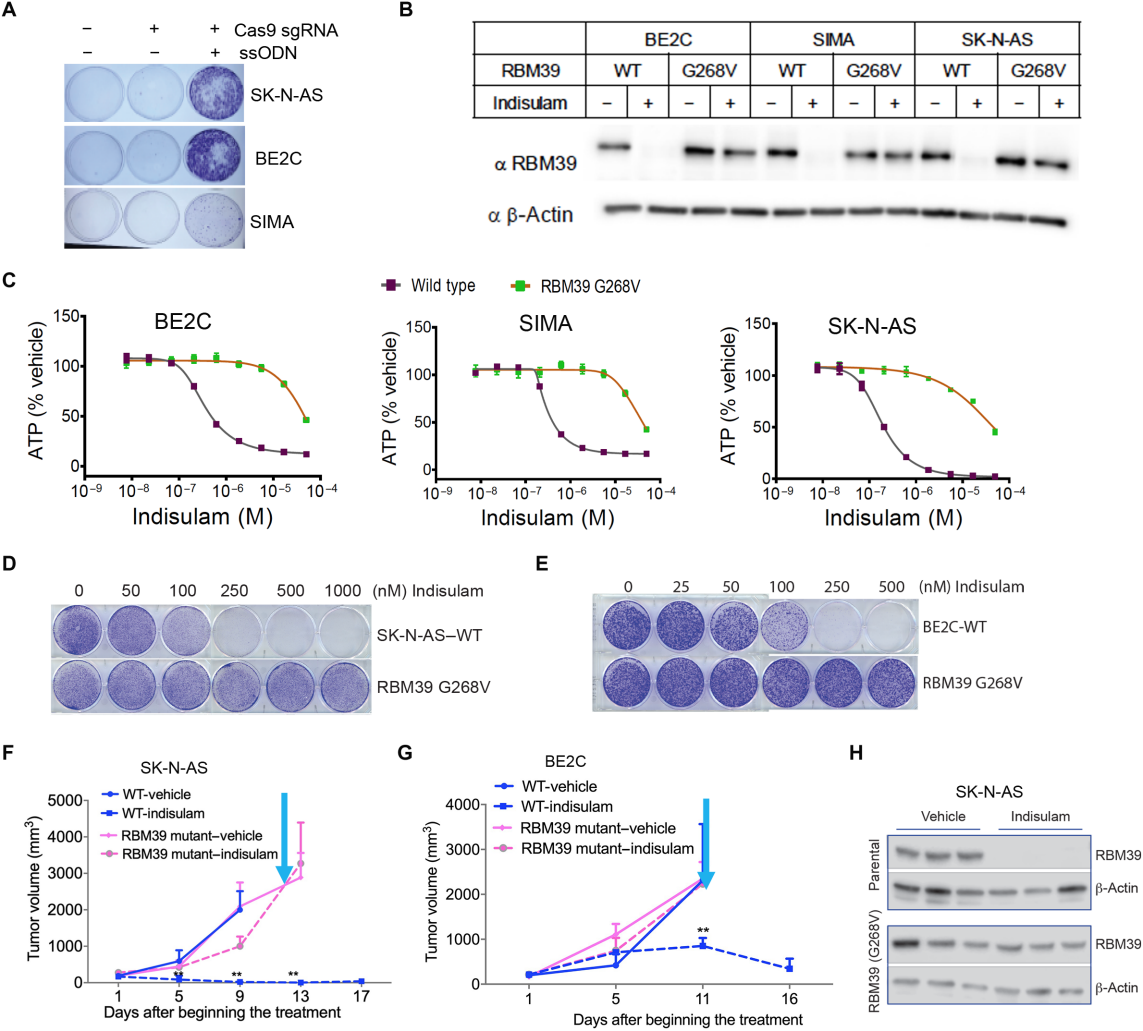
The antitumor effect of indisulam is specifically by targeting RBM39. (**A**) Generation of G268V RBM39 knock-in cell lines. Cells modified with CRISPR-Cas9 and single-stranded oligo (ssODN) carrying the G268V mutation were selected under 2 μM indisulam treatment. Surviving cells were stained with crystal violet. (**B**) Cells with RBM39 G268V mutants generated by CRISPR-Cas9 knock-in were treated with 2 μM indisulam for 18 hours, followed by Western blot analysis. (**C**) Dose-response curve of cell viability assay for cells engineered to express the RBM39 G268V mutant. (**D** and **E**) Colony formation assays for SK-N-AS (D) and BE2C (E) and their RBM39 mutant G268V derivatives that were treated with different concentrations of indisulam. (**F**) Mean tumor volume for SK-N-AS xenografts (vehicle = 3; indisulam = 5) and SK-N-AS with RBM39 G268V knock-in xenografts (vehicle = 5; indisulam = 4) that were treated with indisulam (5 mg/kg), 5 days on and 2 days off for two cycles via tail vein injection. Cyan arrow indicates when therapy was discontinued. (**G**) Mean tumor volume for BE2C xenografts (vehicle = 4; indisulam = 5) and BE2C with RBM39 G268V knock-in xenografts (vehicle = 5; indisulam = 5) that were treated with indisulam (5 mg/kg), 5 days on and 2 days off for two cycles via tail vein injection. Cyan arrow indicates when therapy was discontinued. (**H**) Western blot assessment of RBM39 in SK-N-AS parental and G268V xenografts (vehicle = 3; indisulam = 3) harvested 24 hours after 3-day dosing treatment.

To test whether the antitumor activity of indisulam in neuroblastoma is an RBM39-dependent effect rather than due to off-target activity, we engineered each of three neuroblastoma cell lines (SIMA, BE2C, and SK-N-AS), using CRISPR-Cas9 knock-in, to create indisulam-resistant RBM39 variants (RBM39 G268V) (Fig. 3A). A glycine to valine substitution in RBM39 (G268V) prevents its recruitment to DCAF15 and renders it resistant to degradation without affecting its essential role in pre-mRNA splicing (*23*). G268V-mutant cell lines were resistant to indisulam-mediated RBM39 degradation (Fig. 3B). Moreover, these cells were more than 100-fold resistant to the antiproliferative activity of indisulam (Fig. 3C). RBM39 G268V mutations also rendered neuroblastoma cells resistant to indisulam in colony formation assays (Fig. 3, D and E) and xenograft tumor regression (Fig. 3, F and G). The on-target action of indisulam in tumor cells was further demonstrated by resistance to indisulam-induced RBM39 degradation in vivo (Fig. 3H). On the basis of these results, we concluded that indisulam-mediated degradation of RBM39 is the mechanism of action that accounts for its antitumor effect in neuroblastoma both in culture and in vivo.

### High expression of *DCAF15* in neuroblastoma is correlated with indisulam activity

**Fig. 4.**
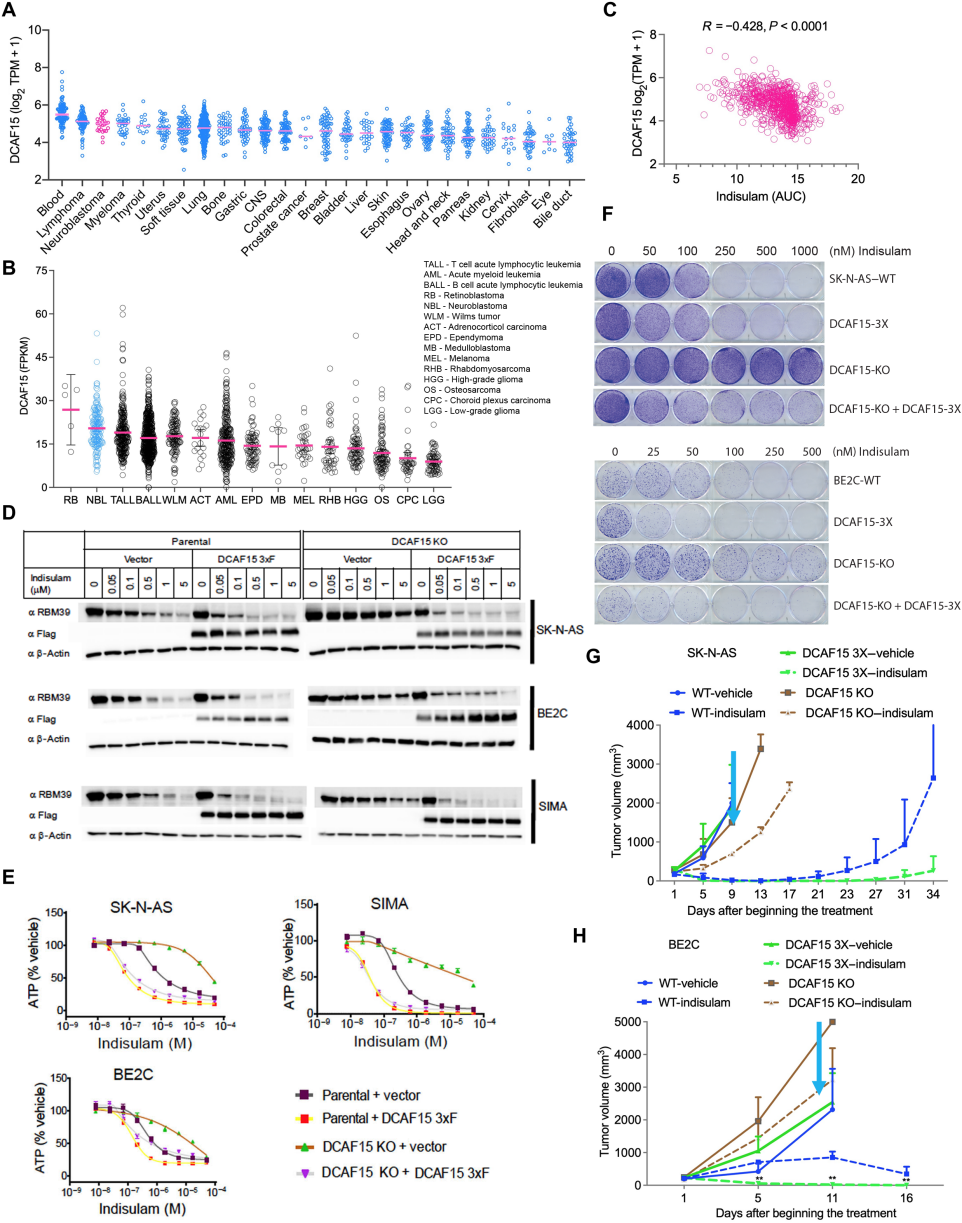
DCAF15 levels are correlated with indisulam efficacy. (**A**) RNA-seq data extracted from the Broad Institute Cancer Cell Line Encyclopedia showed *DCAF15* mRNA expression in different cancer lineages. (**B**) RNA-seq data extracted from the St. Jude Cloud showing *DCAF15* mRNA expression in different pediatric cancer samples (*n* = 2222). (**C**) Spearman correlation shows the correlation of indisulam sensitivity and levels of expression of *DCAF15* across 709 cell lines (*R* = 0.428, *P* < 0.0001). (**D**) BE2C, SIMA, and SK-N-AS cells were engineered to overexpress Flag-tagged DCAF15 (DCAF15 3XF) or DCAF15 KO and the KO rescue cells (DCAF15 KO + DCAF15 3XF), then treated with indicated concentrations of indisulam, and assessed by Western blot analysis with the indicated antibodies. (**E**) Dose-response curve of cell viability assay for engineered cell lines from (D). (**F**) Colony formation assays for SK-N-AS, BE2C, and their derivative lines (D) that were treated with different concentrations of indisulam. (**G** and **H**) Tumor volumes for SK-N-AS (G), BE2C (H), and their derivatives (DCAF15 3XF and DCAF15 KO) that were treated with indisulam (5 mg/kg; *n* = 5 per group) for 5 days on and 2 days off, two cycles via tail injection. The wild-type group data were generated from the same group of mice as used in Fig. 3 (F and G). Cyan arrow indicates when therapy was discontinued.

A prerequisite for indisulam-mediated RBM39 degradation is *DCAF15* expression, and the degree of RBM39 degradation is proportional to the level of *DCAF15* expression (*23*). After establishing the dependency of neuroblastoma on RBM39, we investigated what role *DCAF15* expression levels play in determining the high activity of indisulam in neuroblastoma over other cancer lineages. Among cell lines representing 26 different cancer lineages, neuroblastoma cells expressed relatively high levels of *DCAF15* mRNA, comparable to blood and lymphoma lineages (Fig. 4A and table S11). In agreement, *DCAF15* expression levels are higher in neuroblastoma tumors when compared to multiple adult solid tumors (fig. S11). We further compared *DCAF15* expression between neuroblastomas and other types of pediatric cancers, among 2222 cases analyzed by RNA-seq (St. Jude Cloud) (*33*), and found that neuroblastoma had the second highest levels of *DCAF15*, behind only retinoblastoma (Fig. 4B and table S12).

Relatively high *DCAF15* expression may, in part, explain the exquisite sensitivity of neuroblastoma cancers to indisulam. Consistent with this hypothesis, among 709 different cancer cells in the CTRP, we found a significant correlation between indisulam sensitivity and *DCAF15* expression (Fig. 4C). To directly test whether the antiproliferative effect of indisulam in neuroblastoma cells depends on DCAF15 expression, we engineered neuroblastoma cell lines to either overexpress *DCAF15*, silence *DCAF15*, or rescue *DCAF15* in *DCAF15* knockout (KO) cells (Fig. 4D). The genetic perturbations of *DCAF15* did not affect expression of endogenous RBM39 (fig. S12). However, indisulam-mediated RBM39 degradation did correlate with *DCAF15* levels. *DCAF15* KO reduced degradation, whereas *DCAF15* overexpression enhanced degradation (Fig. 4D). The effects of indisulam on the viability of these cells in vitro mirrored the changes observed with RBM39 degradation (Fig. 4, E and F). The efficacy of indisulam in reducing tumor growth in xenograft studies was higher in cells ectopically expressing *DCAF15* and lower in *DCAF15*-silenced cells (Fig. 4, G and H). Together, these data demonstrate that *DCAF15* expression levels determine the efficacy of indisulam with high levels of *DCAF15* in neuroblastoma conferring more sensitivity to indisulam treatment.

### Indisulam treatment results in significant response in high-risk neuroblastoma models

**Fig. 5.**
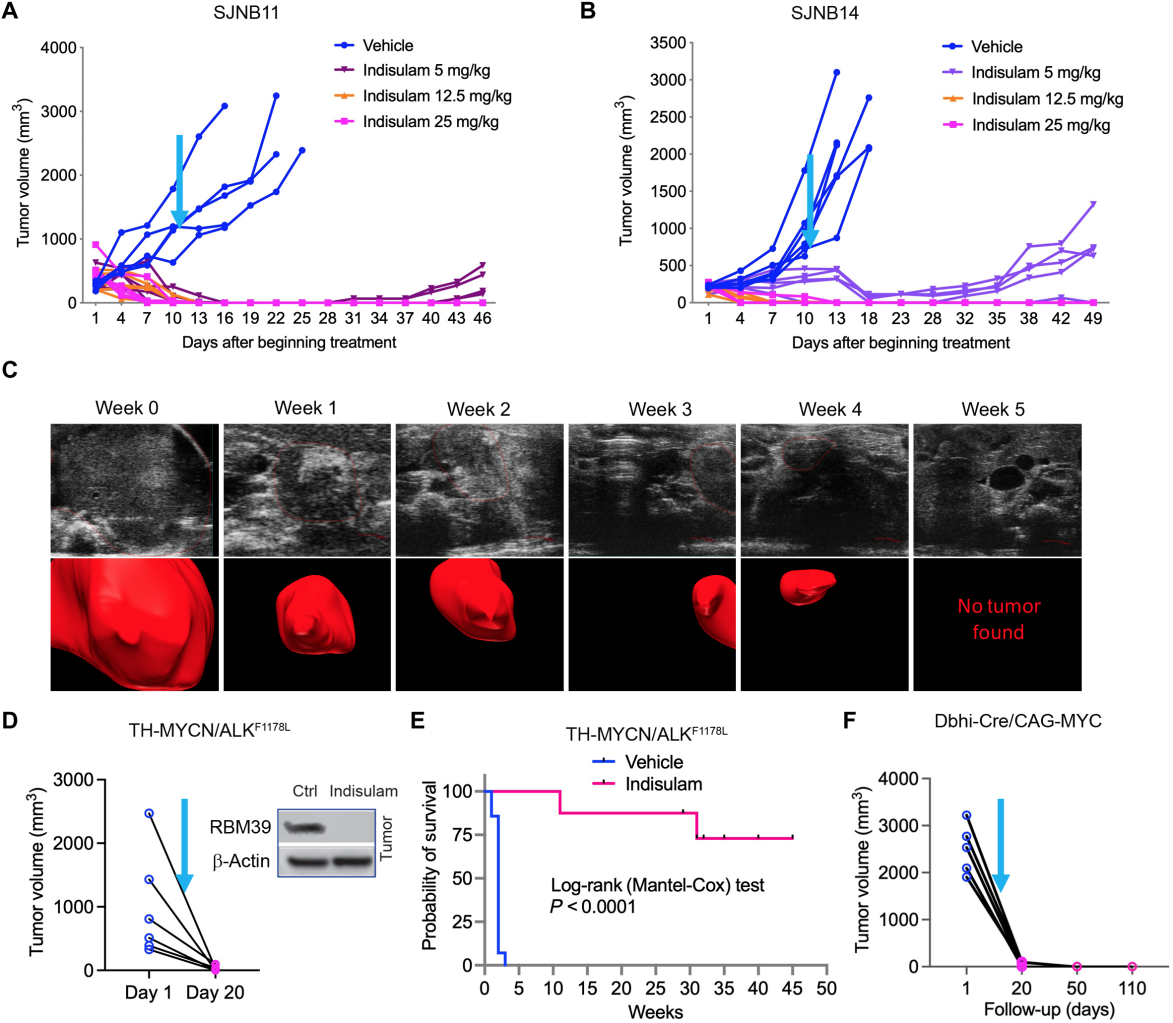
Indisulam affects a significant response in high-risk neuroblastoma models. (**A** and **B**) Individual tumor volumes for SJNB11 and SJNB14 xenografts treated with vehicle or one of three different doses of indisulam, 5, 12.5, and 25 mg/kg, respectively (*n* = 5 per dosing group), 5 days on and 2 days off for two cycles via tail vein injection. Cyan arrow indicates when therapy was discontinued. (**C**) Selected two-dimensional ultrasound images (top row) and volume reconstructions (bottom row) from a representative transgenic *MYCN*/*ALK*^F1178L^ mouse after the standard dose schedule, demonstrating a rapid and sustained response to treatment. (**D**) Individual tumor volume for transgenic *MYCN*/*ALK*^F1178L^ mice that were treated with vehicle (*n* = 10) or indisulam (25 mg/kg; *n* = 6), standard schedule. The inlet shows the target engagement of indisulam by immunoblotting of protein from tumor tissue from one mouse treated with indisulam for 3 days. Cyan arrow indicates when therapy was discontinued. (**E**) Kaplan-Meier curve showing the survival of transgenic MYCN/ALK^F1178L^ mice after treatment. (**F**) Individual tumor volume for syngeneic C-MYC neuroblastoma models that were treated with indisulam (25 mg/kg; *n* = 5), standard schedule. Cyan arrow indicates when therapy was discontinued.

On the basis of above results, we concluded that indisulam-mediated degradation of RBM39 is a viable strategy to target pre-mRNA splicing in MYC-driven neuroblastoma. To further gauge the therapeutic efficacy of indisulam, we administered indisulam to immunodeficient mice harboring xenografts derived from patients with relapsed neuroblastoma, which have never been cultured in vitro, with three different doses of indisulam (5, 12.5, and 25 mg/kg). Indisulam led to a dose-dependent regression of tumor growth, although animals treated with the lowest dose, 5 mg/kg, had evidence of early tumor relapse (Fig. 5, A and B). The comparative studies demonstrate the conservation of alternative (61%) and constitutive (74%) splice junctions in mouse and human genomes, and splicing mechanism is largely conserved between human and mouse (*34*). We therefore hypothesized that mouse neuroblastomas driven by MYC will have a dependency on RBM39 analogous to human neuroblastoma. To test this hypothesis, we evaluated the efficacy of indisulam in an immune-competent, transgenic mouse neuroblastoma model driven by *MYCN* and *ALK^F1178L^* (*TH-MYCN/ALK^F1178L^*) by using ultrasound imaging to quantify tumor response. Human *ALK^F1174L^* (analogous to mouse *ALK^F1178L^*) is the most frequent somatic mutation in neuroblastoma and is associated with *MYCN* amplification, conferring a worse prognosis than *MYCN* amplification alone (*35*). Indisulam led to a complete response shortly after treatment of *TH-MYCN/ALK^F1178L^* mice even when tumor volume at initiation of treatment exceeded 2000 mm^3^ (Fig. 5C). Tumors collected from treated mice before regression displayed a substantial reduction in RBM39 levels, consistent with an on-target effect (Fig. 5C, inlet). This response has been durable for over 50 weeks in four of six mice (Fig. 5, D and E, and fig. S13), despite only having received therapy for 2 weeks. We further tested the efficacy of indisulam in a syngeneic C-MYC neuroblastoma model, in which tumors derived from *Dbh-iCre*/*CAG-C-MYC* mice were engrafted in immune-competent mice of the same genetic background. Indisulam was administered when tumor sizes were between 2000 and 3500 mm^3^. Again, indisulam treatment resulted in durable complete response in all tumors (Fig. 5F). Together, these data reveal that indisulam induces a complete and durable antitumor response in different MYC-driven high-risk neuroblastoma models.

### Indisulam shows no overt toxicity in mouse models

**Fig. 6.**
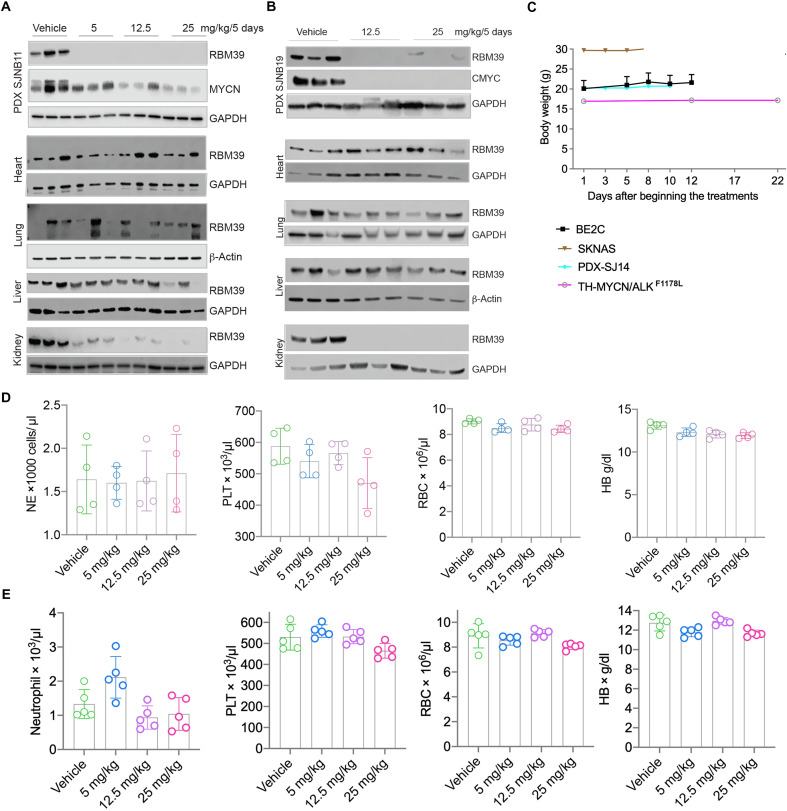
Indisulam shows no overt toxicity in mouse models. (**A**) Western blot assessment of RBM39 in tumor tissues, heart, lungs, kidney, and liver after CB17 SCID mice bearing SJNB11 PDX tumors were treated with vehicle or one of three different doses of indisulam, 5, 12.5, and 25 mg/kg, respectively (*n* = 3 per dosing group), for 5 days. (**B**) Western blot assessment of RBM39 in tumor tissues, heart, lungs, kidney, and liver after CB17 SCID mice bearing SJNB19 PDX (non–*MYCN*-amplified) tumors were treated with vehicle or one of two different doses of indisulam, 12.5 and 25 mg/kg, respectively (*n* = 3 per dosing group), for 5 days. (**C**) Mouse body weight for four neuroblastoma models that were treated with indisulam (25 mg/kg) for 5 days on and 2 days off, two cycles. (**D**) Analysis of neutrophils (NE), platelets (PLT), red blood cells (RBC), and hemoglobin (HB) from CB17 SCID mice bearing SJNB11 PDX that were treated with vehicle or one of three different doses of indisulam, 5, 12.5, and 25, respectively (*n* = 4 per dosing group), for 5 days via tail injection. (**E**) Analysis of neutrophils, platelets (PLT), red blood cells (RBC), and hemoglobin (HB) from CB17 SCID mice bearing SJNB11 PDX that were treated with vehicle or one of three different doses of indisulam, 5, 12.5, and 25 mg/kg, respectively (*n* = 5 per dosing group), for 12 days on and 2 days off, for two cycles via tail injection.

To monitor target engagement in patient-derived xenograft (PDX) mouse models, we used Western blot analysis to determine the levels of RBM39 in tumor tissues as well as normal organs including heart, lungs, liver, and kidney 120 hours following indisulam administration (24 hours after the fourth dosing). In tumors, we observed a reduction in RBM39 at all three doses administered in two different PDX models (Fig. 6, A and B). In contrast, there was no consistent reduction in RBM39 in heart, lungs, and liver, although there was some reduction in the kidney (Fig. 6, A and B), which may be a reflection of *DCAF15* expression levels. Among all tested animals, we did not observe a significant change in mouse body weight during treatment (Fig. 6C). Although a phase 1 study of indisulam showed that nearly all severe adverse events were hematological toxicities (thrombocytopenia, neutropenia, and anemia) (*36*, *37*), we found no substantial changes in blood cell counts (neutrophils, platelets, red blood cells, and hemoglobin) during either the first or second cycle of treatment when using the dosing schedule of 25 mg/kg (Fig. 6, D and E). These data indicate that indisulam efficiently targets RBM39 in tumors but, at least in mice, not in most normal tissues examined.

## DISCUSSION

Spliceosome-mediated pre-mRNA editing is an essential biological process that produces mature RNAs that serve as templates for protein synthesis. Alterations in RNA splicing pathways can lead to dysregulated gene splicing and a tumor-specific dependence (*38*, *39*). The MYC proto-oncogene is an amplifier of transcription of numerous genes including those involved in cell growth, cell cycle, and metabolism. During MYC-driven oncogenic transformation, cells must adapt to the resulting increased burden in RNA processing. Here, we show that pathways involving RNA metabolism and splicing are the most differentially expressed and required in high-risk neuroblastoma. We observed that one of these splicing factors, in particular, RBM39, is essential to RNA splicing in neuroblastoma cells. In genome-wide RNAi screens, suppression of RBM39 had a greater impact on neuroblastoma proliferation than other lineages. The dependency of neuroblastoma on RBM39 is consistent with the concept that RNA splicing may be an “Achilles’ heel” of MYC-driven cancer (*40*). The mechanism for why RBM39 is especially essential in MYC-driven neuroblastoma is an important question to address in the future and has the potential to reveal new targets. We noticed that RBM39 depletion resulted in missplicing of a number of cancer genes that are involved in cell cycle, apoptosis, signal transduction, DNA repair, metabolism, and epigenetic modifications. Missplicing of these candidate genes may explain the greater degree of RBM39 dependence in neuroblastoma than other lineages. Alternatively, these genes could be synthetically lethal with MYC activation in neuroblastoma. The spliceosome involves nearly 300 proteins that form the megadalton complex, which undergoes dynamic assembly and disassembly of at least five subcomplexes in a stepwise manner during pre-mRNA splicing process (*41*, *42*). While RBM39 has been found to physically interact with U2AF65 (*43*, *44*), an important component of subcomplex E, A, and B of spliceosome, it also interacts with numerous splicing factors whose functions remain to be defined (Fig. 1C) (*23*). RBM39 degradation leads to selective effects on pre-mRNA splicing affecting only a subset of genes, and the precise biochemical complex in which RBM39 regulates these events is currently unknown. Defining the other components of this complex mechanism has the potential to unveil more targets in neuroblastoma. Therefore, which step and which subcomplex(es) of spliceosome will be affected by RBM39 depletion and indisulam treatment is a critical question and warrants further studies.

The dependence of neuroblastoma on RBM39 provides a rationale to target the altered splicing machinery in neuroblastoma using an RBM39 degrader as a therapeutic approach. Targeted protein degradation enables conditional and temporal control over the levels of proteins. This approach has emerged as a new therapeutic modality owing to its potential advantages over traditional occupancy-based inhibitors with respect to dosing, side effects, drug resistance, and modulating “undruggable” targets (*45*). Indisulam is one such molecule that recruits RBM39 as a selective neosubstrate to CRL4-DCAF15 E3 ubiquitin ligase, leading to proteasomal degradation (*23*). In this study, we show that indisulam is particularly effective against neuroblastoma in comparison with other cancer lineages. We have observed significant and durable responses to indisulam in multiple high-risk neuroblastoma models including PDXs derived from relapsed patient samples, transgenic *MYCN*/*ALK*^F1178L^ mice, and tumors derived from *C-MYC*–overexpressing mice. We validated the on-target mechanism of indisulam in vitro and in vivo in several ways. First, we showed that cells expressing the RBM39 mutant G268V, which is incapable of binding indisulam but maintains its functional role in the spliceosome, were about 100-fold more resistant to indisulam in vitro than the parental cells and unresponsive to indisulam treatment in vivo. Second, genetic knockdown and pharmacologic degradation of RBM39 induced similar splicing changes, mainly leading to genome-wide skipped exons. Nevertheless, we noticed that the indisulam treatment induced more robust changes in splicing than the shRNA knockdown of *RBM39* (figs. S3, S7, and S10), which could be attributed to several reasons. First, indisulam might have other “off-target” effects. Second, indisulam may more potently destabilize the splicing complexes than shRNA knockdown due to insufficient *RBM39* depletion. Third, the shRNA knockdown effect lagged behind indisulam-mediated degradation because it takes longer time for knockdown to effect in cells.

We further demonstrate that *DCAF15* expression levels correlate with the efficacy of indisulam. The high expression levels of *DCAF15* in neuroblastoma may explain, in combination with the aforementioned dependence on RBM39, why neuroblastoma is exceptionally sensitive to indisulam treatment in comparison with other cancer lineages. Thus, our data support the rationale for patient stratification based on the expression of *DCAF15*, as a biomarker in future clinical trials. The high levels of *DCAF15* and RBM39 dependence in neuroblastoma might make indisulam especially efficacious against this MYC-driven cancer.

A phase 1 clinical study of indisulam showed that thrombocytopenia was the major dose-limiting toxicity followed by neutropenia (*36*). This was confirmed by a phase 2 clinical study showing that nearly all severe adverse events were hematological toxicities (thrombocytopenia, neutropenia, and anemia). The most common grade 3 and 4 nonhematologic toxicities were transient electrolyte abnormalities (*34*). In our pharmacodynamic studies, all PDXs showed substantial reduction of RBM39 in tumor tissues in response to three different indisulam doses. In contrast, there was no comparable reduction of RBM39 in heart, lungs, and liver, although there was some reduction in the kidney, which may be a reflection of higher *DCAF15* expression levels in this tissue. In addition, we observed no whole-body weight loss of treated animals, which is a common indicator of adverse effects, during the treatment course. Whole-blood counts revealed that the three different dosing schedules had no significant adverse effect on the number of neutrophils, platelets, and hemoglobin or red blood cells, in contrast to human patients. The different toxicity profiles may be due to an off-target effect of indisulam in human patients or a mechanism-related effect that is species specific. Although neuroblastoma expresses high levels of *DCAF15* and is highly responsive to indisulam in comparison with other cancer lineages, it might be necessary in the future to stratify patients who are most likely to have the best response. It will be interesting to see whether any genetic surrogates could serve as biomarkers for indisulam treatment. It is worth noting that the neuroblastoma mouse model driven by *MYCN*/*ALK^F1174L^* and *C-MYC* had even better outcomes following indisulam treatment than the PDX and cell line–generated models, which are conducted in immunodeficient mice. This observation requires follow-up investigation because it raises the possibility that indisulam’s efficacy might be enhanced by the immune system.

In clinical trials performed before understanding the mechanism of action of indisulam, only a small population of patients responded to indisulam. However, patients with neuroblastoma were not included in these trials. Here, we show that the RBM39 dependence and high levels of *DCAF15* expression in neuroblastoma account for exceptional efficacy and a high therapeutic index of indisulam in a variety of high-risk neuroblastoma models. Thus, a clinical trial of indisulam for high-risk neuroblastoma is warranted, and preclinical studies are needed to evaluate indisulam in other solid tumors such as ovarian cancers with high *MYCN* levels (*46*).

## MATERIALS AND METHODS

### Pathological information and source of cell lines and patient-derived xenografts

PDX models were propagated in CB17 severe combined immunodeficient (SCID) mice. KELLY, SIMA, BE2C, SK-N-AS, and SK-N-SH were cultured in 1× RPMI 1640 (Corning, 15-040-CV) supplemented with 10% fetal bovine serum (Sigma-Aldrich, F2442), 1% l-glutamine (Corning, A2916801), and 1% penicillin/streptomycin (Gibco, 15140122). CHLA20 was cultured in 1× Iscove’s modified Dulbecco’s medium (Gibco, 12440053) supplemented with 20% fetal bovine serum (Sigma-Aldrich, F2442), 1% l-glutamine (Corning, A2916801), 1% penicillin/streptomycin (Gibco, 15140122), and 1× insulin-transferrin-selenium (Gibco, 41400045). NGP, SKNDZ, CHP212, SK-N-FI, HS68, and BJ cells were cultured in high-glucose Dulbecco’s modified Eagle’s medium (Corning, 15-013-CV) supplemented with penicillin/streptomycin (100 U/ml; Gibco, 15140122), 2 mM l-glutamine (Gibco, A2916801), and 10% fetal bovine serum (FBS) (Sigma-Aldrich, F2442). All cells were maintained at 37°C in an atmosphere of 5% CO_2_. All human-derived cell lines were validated by short tandem repeat (STR) profiling using the PowerPlex 16 HS System (Promega) once a month. In addition, a PCR-based method was used to screen for mycoplasma once a month using the LookOut Mycoplasma PCR Detection Kit (Sigma-Aldrich, MP0035) and JumpStart Taq DNA Polymerase (Sigma-Aldrich, D9307) to ensure that cells were free of mycoplasma contamination.

### Derivation of indisulam-resistant SK-N-AS cells from indisulam-resistant SK-N-AS tumors

The vehicle-treated SK-N-AS tumor (*n* = 3) and indisulam-resistant SK-N-AS tumors (*n* = 3) were excised and placed in a sterile tube containing phosphate-buffered saline (PBS) on wet ice during transport from the animal research facility to the research laboratory. Tumor samples were manually minced using a sterile scalpel and underwent an enzymatic digestion with collagenase IV (2 mg/ml; in 25 ml of RPMI medium) for 1 hour in a 37°C rotor (Robbins Scientific Corporation, model 2000). After digestion, cells were filtered using a 70-μm sterile strainer and cultured in RPMI medium with 10% FBS and 1% penicillin and streptomycin.

### Compound

Indisulam was purchased from MedKoo Biosciences (#201540).

### Antibodies

The following antibodies were used: ß-actin (Sigma-Aldrich, A5441, mouse antibody, 1:5000 dilution), glyceraldehyde-3-phosphate dehydrogenase (GAPDH; Cell Signaling Technology, 5174s, rabbit antibody, 1:5000 dilution), RBM39 (Atlas Antibodies, HPA-001591, rabbit antibody, 1:2000 dilution), c-Myc (Cell Signaling Technology, 5605s, rabbit antibody, 1:2000 dilution), MYCN (Santa Cruz Biotechnology, 53993, mouse antibody, 1:200 dilution), FLAG (Sigma-Aldrich, F1804, mouse antibody, 1:2000 dilution), ATM (D2E2) (Cell Signaling Technology, 2873, rabbit antibody, 1:1000 dilution), EZH2 (D2C9) (Cell Signaling Technology, 5246, rabbit antibody, 1:1000 dilution), CDK4 (D9G3E) (Cell Signaling Technology, 12790, rabbit antibody, 1:1000 dilution), RET (C31B4) (Cell Signaling Technology, 3223, rabbit antibody, 1:1000 dilution), and EED (Millipore 09-774, rabbit antibody, 1:1000 dilution).

### Knock-in of RBM39 G268V allele

Single-guide RNA (sgRNA) targeting RBM39 (5′-gaactgaagatatgcttcgt-3′) was cloned into the pSpCas9(BB)-2A-Puro (PX459) vector (Addgene, 62988). For RBM39 G268V knock-in, SK-N-AS, BE2C, or SIMA cells were transfected (using TransIT-LT1, Mirus) with PX459-RBM39 sgRNA and the single-stranded oligo 5′-GCTGCAGCAATGGCAAACAATTTACAAAAGGGAAGTGCTGGACCTATGAGGCTTTATGTGGGCTCATTACACTTCAACATAACTGAAGATATGCTTCGCGTGATCTTTGAGCCTTTTGGAAGAGTAAGTCCAGGTTCTTCAATGAATCTTCAGTAGGTTGTTGATCTGAGTATAACTACATAATTTCTGTATGTCTTGCT-3′. The oligonucleotide is engineered to introduce a synonymous mutation at R267, allowing us to confirm that the genomic sequence was edited with this template. Afterward, cells were exposed to 2 μM indisulam for 2 weeks to select for cells with RBM39 G268V knock-in. Cells that survived indisulam treatment were recovered and confirmed to have the correct G268V genomic conversion via amplicon sequencing. Amplicon sequencing of PCR products using genomic DNA as template revealed BE2C (62%), SK-N-AS (75%), and SIMA (55%) reads with the repair template sequence, consistent with a heterozygous effect. HCT116 cells expressing RBM39 mutations were previously described by Han *et al.* (*23*).

### CRISPR-mediated inactivation of DCAF15

One independent guide RNA targeting DCAF15 (5′-CGTGTCCCTCAAGAACATTG-3′) was cloned into lentiCRISPRv2 puro (Addgene #98290). Lentiviral packaging of the resulting plasmid was performed by cotransfecting the plasmid with psPAX2 (Addgene, 12260) and pMD2.G (Addgene, 12259) into lenti-X 293T cells. SK-N-AS, BE2C, and SIMA cells were infected with medium collected from transfected lenti-X 293T cells. Cells stably expressing lentiCRISPRv2 with DCAF15 sgRNA were selected with puromycin (2 μg/ml) for 7 to 10 days to enrich for transduced cells.

### Ectopic expression of 3xFLAG-tagged DCAF15

Full-length complementary DNA (cDNA) encoding DCAF15 with a 3xFLAG epitope at the C-terminal end was cloned into pLVX IRES Blast. The PAM (protospacer adjacent motif) site was mutated to prevent Cas9 from editing ectopic expression of 3xFLAG-tagged DCAF15 in SK-N-AS, BE2C, and SIMA cells generated by CRISPR-mediated deactivation of DCAF15. Lentiviral packaging of the resulting plasmid was performed by cotransfecting with psPAX2 (Addgene, 12260) and pMD2.G (Addgene, 12259) into lenti-X 293T cells. The medium collected from transfected lenti-X 293T cells was used to infect SK-N-AS, BE2C, and SIMA cells. Cells stably expressing 3xFLAG-tagged DCAF15 were selected for 7 to 10 days with blasticidin (10 μg/ml). HCT116 cells were described by Han *et al.* (*23*). Cells were split each into one six-well plate for an 18-hour incubation with indisulam at doses 0 [dimethyl sulfoxide (DMSO)], 0.05, 0.1, 0.5, 1, and 5 μM to validate for 3xFLAG-tagged DCAF15 and RBM39 degradation via Western blot.

### Sanger DNA sequencing of RBM39

Total RNA was isolated from tissue samples as per the manufacturer’s instructions of the RNeasy Mini Kit (Qiagen, catalog no. 74106). cDNA was prepared from 1 μg of total RNA using a cDNA synthesis kit according to the manufacturer’s protocol (Thermo Fisher Scientific, catalog no. 18091050). RBM39 gene was amplified by PCR using the cDNA as the template along with the following primers (forward: 5′-atttctagagccaccatggcagacgatattgatattgaagcaatgc-3′; reverse: 5′-attggatcctcatcgtctacttggaaccagtagc-3′) using the Phusion High Fidelity PCR Kit (catalog no. E0553S). The resultant PCR product was gel-purified using a Qiagen gel extraction kit (Qiagen, catalog no. 28706) according to the manufacturer’s protocol. The DNA was sent for Sanger sequencing at Hartwell Center in St. Jude using the following primers: primer 288F, ACAGAAGTCCTTACTCCGGACC; primer 388R, ACTTTTGCTTCGGGAACGTCG;

primer 602F, GTCGATGTTAGCTCAGTGCCTC. Primer 602F is used to detect the RBM39 mutation in the RRM2 motif.

### Lentiviral packaging of shRNA

The TRC (The RNAi Consortium) lentiviral-based shRNA knockdown plasmids for RBM39 were purchased from Horizon Discovery (shRBM39#188, RHS3979-201752797; shRBM39#190, RHS3979-201752799). The lentiviral shRBM39 and shControl (pLKO.1) particles were packaged by Vector Lab at St. Jude. Briefly, human embryonic kidney (HEK) 293T cells were transfected with shRNA constructs and helper plasmids (pCAG-kGP1-1R, pCAG4-RTR2, and pHDM-G). The 48- and 72-hour posttransfection replication-incompetent lentiviral particles were harvested and transduced into cells with polybrene (8 μg/ml). Forty-eight hours later, puromycin (1 μg/ml) was added for selection for additional 48 hours before injection into mice or immunoblotting.

### SDS–polyacrylamide gel electrophoresis and Western blot

Cells were washed twice with ice-cold PBS and directly lysed on ice with 2× sample loading buffer [0.1 M tris HCl (pH 6.8), 200 mM dithiothreitol, 0.01% bromophenol blue, 4% SDS, and 20% glycerol]. On ice, cell lysates were sonicated once with a 5-s bursts at 40% amplitude output (Sonics, VIBRA CELL) followed by 25-min heating at 95°C. After the cell lysates were centrifuged at 13,000*g* at room temperature for 2 min, 10 to 20 μl of the cell lysates were separated on 4 to 15% Mini-PROTEAN TGX Stain-Free Protein Gels from Bio-Rad and transferred to methanol-soaked polyvinylidene difluoride membranes (Millipore). Lysates for RBM39 G268V mutant cell lines and DCAF15 genetically modified cells were generated as previously described (*23*). Membranes were blocked in PBS buffer supplemented with 0.1% Tween 20 and 5% skim milk (PBS-T) and incubated for 1 hour at room temperature under gentle horizontal shaking. Membranes were incubated overnight at 4°C with the primary antibodies. The next day, membranes were washed three times (for 5 min) with PBS-T at room temperature. Protected from light, membranes were then incubated with goat anti-mouse or goat anti-rabbit horseradish peroxidase–conjugated secondary antibodies (1:5000) for 1 hour at room temperature, followed by three 5-min washes with PBS-T at room temperature. Last, membranes were incubated for 1 min at room temperature with SuperSignal West Pico PLUS Chemiluminescent Substrate (Thermo Fisher Scientific, 34580), and the bound antigen-antibody complexes were visualized using the Odyssey Fc Imaging System (LI-COR Corp., Lincoln, NE).

### Crystal violet staining

After removing medium, cells were washed with Dulbecco’s PBS (DPBS) without calcium or magnesium (DPBS, Lonza) and treated with 4% formaldehyde in PBS [paraformaldehyde (PFA)] for 20 min. Once PFA was removed, cells were stained with 0.1% crystal violet stain for 1 hour.

### Cell viability assay

Twelve-point dose responses were performed on 96-well assay plates with cell plating (1500 to 4000 cells per well) on day 1, compound addition on day 2, and survival measurement on day 5. Compounds were diluted in DMSO before adding to the cells. Final DMSO concentration was 0.5%. Cell survival assay was performed using a CellTiter-Glo Luminescent Cell Viability Assay kit (Promega) that measures cellular adenosine triphosphate (ATP) content. Luminescence was recorded with an EnVision multimode plate reader (PerkinElmer). Median inhibitory concentration (IC_50_) was determined with GraphPad Prism using baseline correction (by normalizing to DMSO control), the asymmetric (five parameter) equation, and least-squares fit.

### PrestoBlue assay

Cell viability was determined by PrestoBlue Cell Viability Reagent (Invitrogen, catalog no. A13262) according to the manufacturer’s protocol. Briefly, cells were plated in white 96-well plates (PerkinElmer, catalog no. 6005680) at a density of 1000 to 3000 cells per well and incubated at 37°C with 5% CO_2_ for 24 hours. Afterward, cells were treated with drugs at different final concentrations (10,000, 500, 250, 50, 25, 12.5, 5, 2.5, 0.5, 0.05, and 0 nM) for 5 days. Each concentration was tested in eight replicates. After completion of the treatment period, 10 μl of PrestoBlue reagent was added to each well and plates were incubated at 37°C with 5% CO_2_ for 30 min. Then, fluorescence (560-nm excitation/590-nm emission) was measured using a BioTek Synergy H1 microplate reader. GraphPad Prism was used to plot the curves and calculate the IC_50_ values.

### Cell cycle analysis

Cells (100, 000 per well) were plated in six-well culture plates and, next day, treated with indisulam for 48 hours. Cells were trypsinized and centrifuged at 1000 rpm for 5 min at 4°C and washed once with ice-cold PBS. The samples were then resuspended in the propidium iodide solution [propidium iodide (0.05 mg/ml), 0.1% sodium citrate, and 0.1% Triton X-100] and incubated at room temperature for 20 min. The cells were subjected to flow cytometric analysis by using an LSR II (BD Biosciences, CA, USA) flow cytometer, and the data were analyzed using BD FACSDiva (BD Biosciences, CA, USA).

### Differential gene expression and GSEA for RNA-seq experiments

Total RNA from cells and tumor tissues was performed using the RNeasy Mini Kit (Qiagen) according to the manufacturer’s instructions. Paired-end sequencing was performed using the High-Seq platform with 100–base pair (bp) read length. Total stranded RNA-seq data were processed by the internal AutoMapper pipeline. Briefly, the raw reads were first trimmed (Trim-Galore version 0.60) and mapped to human genome assembly (GRCh38) (STAR v2.7), and then the gene-level values were quantified (RSEM, v1.31) on the basis of GENCODE annotation (v31). Low-count genes were removed from analysis using a CPM (counts-per-million) cutoff corresponding to a count of 10 reads and only confidently annotated (level 1 and 2 gene annotation), and protein-coding genes are used for differential expression analysis. Normalization factors were generated using the TMM (Trimmed Mean of the M-values) method, counts were then transformed using Voom, and transformed counts were analyzed using the lmFit and eBayes functions (R limma package version 3.42.2). The significantly up- and down-regulated genes were defined by at least twofold changes and adjusted *P* value of <0.05. Then, GSEA was carried out using gene-level log_2_ fold changes from differential expression results against gene sets in the Molecular Signatures Database (MSigDB 6.2) (gsea2 version 2.2.3) for the data from GSE62564.

### RNA splicing analysis

After mapping RNA-seq data, rMATS v4.1.0 was used for RNA alternative splicing analysis by using the mapped BAM files as input. Specifically, five different kinds of alternative splicing events were identified, i.e., skipped exon, alternative 5′ splicing site, alternative 3′ splicing site, mutually exclusive exon, and intron retention. To keep consistent, the same GTF (General Feature Format) annotation reference file for mapping was used for rMATS. For stranded RNA-seq data, the argument “--libType fr-firststrand” was applied. To process reads with variable lengths, the argument “--variable-read-length” was also used for rMATS. To select statistically significantly differential splicing events, the following thresholds were used: false discovery rate (FDR) < 0.05 and absolute value of IncLevelDifference > 0.1. For visualization, the Integrated Genomic Viewer (IGV) Genome Browser was used to show the sashimi plots of splicing events. To investigate the genome-wide correlations of differential splicing between two genotypes (e.g., shRNA knockdown of RBM39 and indisulam-mediated degradation of RBM39 in cells), we extracted splice junctions for all samples of both genotypes of interest from the STAR (*47*) output files suffixed with “SJ.out.tab,” which contain high-confidence collapsed splice junctions. Only those unique mapped reads crossing the junctions were considered. By extracting the union of the unique junction positions, we constructed a unified junction-read feature vector for each sample. Then, we normalized the junction-read vectors of each sample by their corresponding total read counts and multiplied them by 10^6^. Next, we averaged the junction-read vectors for samples of the same genotype. We further excluded those junctions for which the total normalized junction-read counts were smaller than 10. Then, we calculated the Pearson correlation coefficients for each pair of genotypes. The two-sided *t* tests were used to compute the significant levels of the correlations by the function chart.Correlation() in R.

To investigate the genome-wide correlations of differential splicing events between shRNA knockdown of RBM39 and indisulam treatment in different cell lines, we first used rMATS to find the differential alternative splicing events for each pair of comparisons in five splicing categories including exon skipping, mutually exclusive exons, alternative 3′ splicing site, alternative 5′ splicing site, and intron retention. Then, we selected the significant splicing events by using the criteria of FDR < 0.05 and abs(IncLevelDifference) > 0.1. Next, on the basis of these different splicing events, we used Venn diagrams to show their common and unique splicing events in the five splicing categories mentioned above.

### RNA extraction and RT-PCR for RBM39

RNA was extracted using the RNeasy Plus Mini Kit (Qiagen, reference no. 74136) following the manufacturer’s protocol. cDNA was prepared in 20-μl reaction from 500 ng of total RNA using the Superscript IV First Strand Synthesis System (Invitrogen, reference no. 1809105). Real-time PCRs were run in triplicates (*n* = 3) in the 7500 Real-Time PCR System by Applied Biosystems (Thermo Fisher Scientific) using Power SYBR Green PCR Master Mix (Applied Biosystems, reference no. 4367660). ΔΔ*C*_T_ methods were applied to analyze the results. The following primers were used to perform the quantitative real-time PCR: GAPDH (forward, AACGGGAAGCTTGTCATCAATGGAAA; reverse, GCATCAGCAGAGGGGGCAGAG) and RBM39 (forward, AGATGGACAACTGCCTCATTAC; reverse, GCCTCCCAGTGTTCACATATAC).

### RT-PCR for detection of splicing events

For pharmacologic inhibition, BE2C and SKNAS cells were treated with DMSO and indisulam (250 and 500 nM) for 48 hours. For genetic inhibition, BE2C and SKNAS cells were transduced with shCtrl, shRBM39 (#188), and shRBM39 (#190) on day 1 and selected with puromycin (2 μg/ml) on day 2 for 24 hours. Total RNA was extracted after 48 hours using the RNeasy Kit (Qiagen). cDNA was synthesized using the SuperScript IV Reverse Transcriptase Kit (Thermo Fisher Scientific, catalog no. 18091050). RT-PCR was performed using the Phusion High Fidelity PCR Kit (New England BioLabs, catalog no. E0553) using cDNA as the template DNA. The PCR products were run on a 2% agarose gel and scanned using the Odyssey Fc Imaging System Fc (LI-COR Biotechnology). The following PCR primers were used: EZH2 (forward, 5′-TGGGCTGCACACTGCAGAAA-3′; reverse, 5′-ACACTCTCGGACAGCCAGGT-3′), NTRK1 (forward, 5′-AAGGTCCAGGTGCCCAATGC-3′; reverse, 5′-ACCGGGTCTCCAGATGTGCT-3′), CDK4 (forward, 5′-GCCTGTGTCTATGGTCGGGC-3′; reverse, 5′-TCCCGGTCAGTTCGGGATGT-3′), and KIF20B (forward, 5′-AGGTGAAAGGTTAAGAGAGACTGGG-3′; reverse, 5′-GCTCCTCAACCAAATCCTCGT-3′).

### Assay for transposase-accessible chromatin using sequencing

Library preparations for ATAC-seq (assay for transposase-accessible chromatin using sequencing) were based on a published protocol with minor modifications (*48*, *49*). Briefly, freshly cultured SKNAS cells (100,000 per sample, naïve and two resistant ones) were harvested and washed with 150 μl of ice-cold DPBS containing protease inhibitor (PI). Nuclei were collected by centrifugation at 500*g* for 10 min at 4°C after cell pellets were resuspended in lysis buffer [10 mM tris-Cl (pH 7.4), 10 mM NaCl, and 3 mM MgCl_2_ containing 0.1% NP-40 and PI]. Nuclei were incubated with Tn5 transposon enzyme in transposase reaction mix buffer (Illumina) for 30 min at 37°C. DNAs were purified from the transposition sample by using a Mini-Elute PCR purification kit (Qiagen, 28004) and measured by Qubit. PCR was performed to amplify with High-Fidelity 2× PCR Master Mix [72°C/5 min + 98°C/30 s + 12 × (98°C/10 s + 63°C/30 s + 72°C/60 s) + 72°C/5 min]. The libraries were purified using a Mini-Elute PCR purification kit (Qiagen). ATAC-seq libraries were pair-end–sequenced on HiSeq4000 (Illumina) in the Hartwell Center at St. Jude Children’s Research Hospital, Memphis, TN, USA.

### ATAC-seq alignment, peak calling, and annotation

The ATAC-seq raw reads were aligned to the human reference genome (hg19) using BWA (*50*) and then marked duplicated reads with Picard (version 1.65), with only high-quality reads kept by samtools (version 1.3.1, parameter “-q 1 -F 1024”) (*51*). Reads mapping to mitochondrial DNA were excluded from the analysis. All mapped reads were offset by +4 bp for the + strand and −5 bp for the – strand (*48*). Peaks were called for each sample using MACS2 (*52*) with parameters “-q 0.01 –nomodel –extsize 200 –shift 100.” Peaks were merged for the same cell types using BEDtools (*53*). Peak annotation was performed using HOMER (*54*). All sequencing tracks were viewed using IGV 2.3.82 (*55*).

### Data mining

*DCAF15* expression data from various cancer lineages were downloaded from CCLE RNA-seq (https://portals.broadinstitute.org/ccle). *DCAF15* expression data from various tumor tissues were downloaded from R2 program (https://hgserver1.amc.nl/cgi-bin/r2/main.cgi), and *DCAF15* expression data from RNA-seq data of various pediatric cancer tissues were downloaded from St. Jude Cloud (https://pecan.stjude.cloud/). The expression of *RBM39* and clinical survival data were retrieved using R2 program using the dataset [GSE12460 (*56*), GSE7529, and GSE62564]. PRISM data, CTRP data, and RNAi data were downloaded from DepMap database (https://depmap.org/portal/). The epigenetic landscape of RBM39 genomic locus was downloaded from epigenetic browser of St. Jude Cloud (https://pecan.stjude.cloud/proteinpaint/study/mycn_nbl_2018).

### Pathway network analysis

The 1585 essential fitness genes to neuroblastoma cell survival (*n* = 7, dependency score > 40%) identified through genome-wide CRISPR-Cas9 library screen by Sanger institute (*24*) were uploaded into STRING program (https://string-db.org) for network interaction analysis with confidence threshold 0.15. The resulting network was then uploaded into Cytoscape program (*57*), followed by gene ontology analysis using the BiNGO plugin program (*58*).

### Analysis of PRISM data

We loaded the PRISM Repurposing 19Q4 Drug Sensitivity Primary Screen Replicate Collapsed log_2_ fold change data (primary-screen-replicate-collapsed-logfold-change.csv, DepMap.org). The log_2_ fold change is quantified with respect to DMSO and is a measure of cell viability in response to the chemical perturbations. Cell lines containing the string “FAILED_STR” (e.g., ACH-000010_FAILED_STR) were removed, leaving 568 cell lines and 4686 compounds. Twelve of these cell lines are neuroblastoma lineage. Not all compounds have data available for the 568 cell lines. In the case of indisulam, data for 5 neuroblastoma cell lines and 536 non-neuroblastoma cell lines are available.

### Differential sensitivity analysis of neuroblastoma cell lines

For each compound in the primary screen, we calculate the mean log_2_ fold change of neuroblastoma cell lines minus the mean of log_2_ fold change for non-neuroblastoma cell lines. To calculate *P* value of mean difference, we conducted a permutation test (i.e., Monte Carlo sampling) in which we randomly sampled five values from the indisulam log_2_ fold change data and then took the difference in means of those 5 against the remaining 536 values. We randomly sampled in this manner 10^5^ times. On the basis of the distribution of these 10^5^ means, we concluded that indisulam mean difference of −1.30 has a *P* value less than 0.003 and was highly statistically significant. We performed the same calculation for the PRISM Repurposing 19Q4 Drug Sensitivity Secondary Screen AUC data (secondary-screen-dose-response-curve-parameters.csv). Again, we removed any cell lines in the data labeled with FAILED STR. For a given cell line and compound, there are AUC data from multiple screens. The documentation provided with the data (the README file) recommends using screen “MTS010” when available. The figures contain data from only this screen. Restricting to screen MTS010, we have 473 cell lines and 147 compounds, although any given compound may not have coverage across all 473 cell lines. In the case of indisulam, data were available for 4 neuroblastoma cell lines and 438 non-neuroblastoma cell lines. For each compound in the MTS010 screen, we calculated the mean AUC of neuroblastoma cell lines minus the mean AUC of non-neuroblastoma cell lines. We conducted a permutation test to calculate *P* value of mean difference AUC in which we randomly sampled four values from the indisulam AUC data and then took the difference in means of those 4 against the remaining 438 values. We randomly sampled in this manner 10^5^ times. On the basis of the distribution of these 10^5^ means, we conclude that indisulam mean difference AUC of −0.226 has a *P* value less than 0.001 and is highly statistically significant.

### shRNA xenograft studies

Neuroblastoma cells were transduced with shRNA lentiviral particles targeting RBM39. Forty-eight hours later, puromycin (1 μg/ml) was added for selection for additional 72 hours (BE2C) or 48 hours (SKNAS, KELLY, and SIMA). Cancer cells (2.2 × 10^6^ for BE2C, 5 × 10^6^ for SIMA and SKNAS, and 2.5 × 10^6^ for KELLY) were mixed with Matrigel (1:1 ratio in volume) and subcutaneously injected into the flank sites of NSG (NOD scid gamma) mice. Mice were sacrificed when they reached the humane endpoint for BE2C (day 12), SKNAS (day 14), SIMA (day 8), and KELLY (day 8) from the day measurement was started (day 1). Tumors were measured by using electronic calipers, and volumes were calculated as width π/6 × *d*^3^, where *d* is the mean of two diameters taken at right angles.

### Transgenic TH-MYCN/ALK^F1178L^ mouse model

This model was provided by P. Murray, P. Thomas, and L.-A. Van de Velde, the details of which have been reported (*59*). The St. Jude Children’s Research Hospital Institutional Animal Care and Use Committee approved all studies performed. Following genotyping, *TH-MYCN*/*ALK*^F1178L^ mice were gender-segregated and assigned to imaging groups. Inclusion criteria were the presence of the *TH-MYCN* allele, heterozygosity for one *ALK*^F1178L^ allele, and an age range from 1 to 3 weeks postweaning. Either gender was used and noted. Criteria for analysis were the presence or absence of an ultrasound-detectable tumor (quantified by staff not directly involved in this study) at any point within a 7-week period, where mice were imaged once per week.

### Ultrasound imaging of TH-MYCN/ALK^F1178L^ mouse

Fur was removed from the ventral side of each animal using Nair. Technicians in the St. Jude Center for In Vivo Imaging and Therapeutics performed ultrasound scanning on mice weekly using VEVO-2100 and determined tumor volumes using VevoLAB 3.0.0 software. All ultrasound data were acquired in a blinded fashion.

### C-MYC syngeneic neuroblastoma model

Primary tumors derived from *Dbh-iCre*; *CAG-MYC*^LSL-KI/LSL-KI^ were provided by R. Wang (this mouse model will be reported separately), which were subcutaneously engrafted in CB57BL6 mice. When tumor sizes reached about 2000 to 3500 mm^2^ but below 20% of mouse body weight, indisulam treatment was started.

### Indisulam efficacy studies in in vivo models

All murine experiments were done in accordance with a protocol approved by the Institutional Animal Care and Use Committee of St. Jude Children’s Research Hospital. Subcutaneous xenografts were established in CB17 SCID mice (CB17 scid, Taconic) or NOD.Cg-Prkdcscid Il2rgtm1Wjl/SzJ (NSG; The Jackson Laboratory) mice by implanting 5 × 10^6^ cells in Matrigel. We used both genders in NSG or transgenic TH-MYCN/ALK^F1178L^ mice models to exclude sex bias. Tumor measurements were done weekly using electronic calipers, and volumes were calculated as width π/6 × *d*^3^, where *d* is the mean of two diameters taken at right angles. Mice were randomly assigned to experimental groups at 4 to 6 weeks of age. A 2-week treatment commenced when the implanted tumor volume reached above 200 mm^3^. As a standard dosing schedule, indisulam (25 mg/kg) was administered via tail vein injection, once daily, for 5 days on and 2 days off. Tumor volume and body weight were measured twice weekly. Mice were euthanized when the tumor volume reached 20% of the body weight or the mice became moribund. Tumor response: For individual mice, progressive disease (PD) was defined as <50% regression from initial volume during the study period and >25% increase in initial volume at the end of study period. Stable disease (SD) was defined as <50% regression from initial volume during the study period and ≤25% increase in initial volume at the end of the study. Partial response (PR) was defined as a tumor volume regression of ≥50% for at least one time point but with measurable tumor (≥0.10 cm^3^). Complete response (CR) was defined as a disappearance of measurable tumor mass (<0.10 cm^3^) for at least one time point.

### Pharmacodynamic studies

Tumor-bearing mice (SJNB11 and SJNB19 PDXs) were treated with indisulam (5, 12.5, and 25 mg/kg) for five consecutive days via tail injection. At the end of the experiment, mice were euthanized by CO_2_. Tumor and organ samples were excised and immediately snap-frozen in liquid nitrogen and preserved at −80°C until further use. Tissue lysates were made using 2× lysis buffer [1 M tris-HCl (pH 6.8), 10% SDS, glycerol, β-mercaptoethanol, and bromophenol blue]. Briefly, a 5-mm^3^ piece of tissue was added to 500 μl of 2× lysis buffer, homogenized for 30 s (PRO200, Biogen, USA), heated at 95°C, and centrifuged at 12,000 rpm for 5 min at 4°C. Supernatant was further diluted 5×, sonicated for 30 s, and heated at 95°C. The sample was centrifuged at 13,000 rpm for 5 min and used as whole-tissue protein lysate.

### Complete blood counting

Complete blood count was analyzed with the Forcyte analyzer hematology machine, according to the manufacturer’s instructions. Briefly, blood was collected in Eppendorf tubes containing 10 μl of 10% EDTA via retroorbital bleed using 200-μl heparinized capillary tubes (catalog no. 22-362-566, Fisher brand). Blood samples were processed within 2 hours to avoid hemolysis. The number of leukocytes (white blood cell), erythrocytes (red blood cell), lymphocytes, neutrophils, monocytes, eosinophils, and platelets was counted. Proprietary lysing agent was added to liberate hemoglobin and ultimately convert it to cyanmethemoglobin to calculate the value.

### Statistical analysis

Wilcoxon rank sum test (two-sided) was used to compare the volume between two groups at every time point. *P* values across multiple time points were adjusted for multiple comparison using the Benjamin-Hochberg method. Kaplan-Meier survival analyses were used with log-rank (Mantel-Cox) method in Prism program.

## Notes

View/request a protocol for this paper from *Bio-protocol*.

## References

[1] K. K. Matthay, J. M. Maris, G. Schleiermacher, A. Nakagawara, C. L. Mackall, L. Diller, W. A. Weiss, Neuroblastoma. Nat. Rev. Dis. Primers 2, 16078 (2016). 27830764 10.1038/nrdp.2016.78

[2] E. Suh, K. L. Stratton, W. M. Leisenring, P. C. Nathan, J. S. Ford, D. R. Freyer, J. L. Mc Neer, W. Stock, M. Stovall, K. R. Krull, C. A. Sklar, J. P. Neglia, G. T. Armstrong, K. C. Oeffinger, L. L. Robison, T. O. Henderson, Late mortality and chronic health conditions in long-term survivors of early-adolescent and young adult cancers: A retrospective cohort analysis from the Childhood Cancer Survivor Study. Lancet Oncol. 21, 421–435 (2020). 32066543 10.1016/S1470-2045(19)30800-9 PMC7392388

[3] P. C. Nathan, K. K. Ness, M. L. Greenberg, M. Hudson, S. Wolden, A. Davidoff, C. Laverdiere, A. Mertens, J. Whitton, L. L. Robison, L. Zeltzer, J. G. Gurney, Health-related quality of life in adult survivors of childhood Wilms tumor or neuroblastoma: A report from the childhood cancer survivor study. Pediatr. Blood Cancer 49, 704–715 (2007). 16830322 10.1002/pbc.20949

[4] M. Eilers, R. N. Eisenman, Myc’s broad reach. Genes Dev. 22, 2755–2766 (2008). 18923074 10.1101/gad.1712408 PMC2751281

[5] Z. E. Stine, Z. E. Walton, B. J. Altman, A. L. Hsieh, C. V. Dang, MYC, metabolism, and cancer. Cancer Discov. 5, 1024–1039 (2015). 26382145 10.1158/2159-8290.CD-15-0507 PMC4592441

[6] J. H. Schulte, A. Eggert, Neuroblastoma. Crit. Rev. Oncog. 20, 245–270 (2015). 26349419 10.1615/critrevoncog.2015014033

[7] W. C. Gustafson, W. A. Weiss, Myc proteins as therapeutic targets. Oncogene 29, 1249–1259 (2010). 20101214 10.1038/onc.2009.512 PMC2904682

[8] D. S. Rickman, J. H. Schulte, M. Eilers, The expanding world of N-MYC-driven tumors. Cancer Discov. 8, 150–163 (2018). 29358508 10.1158/2159-8290.CD-17-0273

[9] M. Huang, W. A. Weiss, Neuroblastoma and MYCN. Cold Spring Harb. Perspect. Med. 3, a014415 (2013). 24086065 10.1101/cshperspect.a014415 PMC3784814

[10] M. W. Zimmerman, Y. Liu, S. He, A. D. Durbin, B. J. Abraham, J. Easton, Y. Shao, B. Xu, S. Zhu, X. Zhang, Z. Li, N. Weichert-Leahey, R. A. Young, J. Zhang, A. T. Look, *MYC* drives a subset of high-risk pediatric neuroblastomas and is activated through mechanisms including enhancer hijacking and focal enhancer amplification. Cancer Discov. 8, 320–335 (2018). 29284669 10.1158/2159-8290.CD-17-0993 PMC5856009

[11] C. J. David, M. Chen, M. Assanah, P. Canoll, J. L. Manley, HnRNP proteins controlled by c-Myc deregulate pyruvate kinase mRNA splicing in cancer. Nature 463, 364–368 (2010). 20010808 10.1038/nature08697 PMC2950088

[12] T. Y.-T. Hsu, L. M. Simon, N. J. Neill, R. Marcotte, A. Sayad, C. S. Bland, G. V. Echeverria, T. Sun, S. J. Kurley, S. Tyagi, K. L. Karlin, R. Dominguez-Vidaña, J. D. Hartman, A. Renwick, K. Scorsone, R. J. Bernardi, S. O. Skinner, A. Jain, M. Orellana, C. Lagisetti, I. Golding, S. Y. Jung, J. R. Neilson, X. H.-F. Zhang, T. A. Cooper, T. R. Webb, B. G. Neel, C. A. Shaw, T. F. Westbrook, The spliceosome is a therapeutic vulnerability in MYC-driven cancer. Nature 525, 384–388 (2015). 26331541 10.1038/nature14985 PMC4831063

[13] C. M. Koh, M. Bezzi, D. H. P. Low, W. X. Ang, S. X. Teo, F. P. H. Gay, M. Al-Haddawi, S. Y. Tan, M. Osato, A. Sabò, B. Amati, K. B. Wee, E. Guccione, MYC regulates the core pre-mRNA splicing machinery as an essential step in lymphomagenesis. Nature 523, 96–100 (2015). 25970242 10.1038/nature14351

[14] K. Iwai, M. Yaguchi, K. Nishimura, Y. Yamamoto, T. Tamura, D. Nakata, R. Dairiki, Y. Kawakita, R. Mizojiri, Y. Ito, M. Asano, H. Maezaki, Y. Nakayama, M. Kaishima, K. Hayashi, M. Teratani, S. Miyakawa, M. Iwatani, M. Miyamoto, M. G. Klein, W. Lane, G. Snell, R. Tjhen, X. He, S. Pulukuri, T. Nomura, Anti-tumor efficacy of a novel CLK inhibitor via targeting RNA splicing and MYC-dependent vulnerability. EMBO Mol. Med. 10, e8289 (2018). 29769258 10.15252/emmm.201708289 PMC5991599

[15] F. Salvador, R. R. Gomis, CLK2 blockade modulates alternative splicing compromising MYC-driven breast tumors. EMBO Mol Med. 10, –e9213 (2018). 10.15252/emmm.201809213 PMC5991597 29789342

[16] G. K. Alderton, MYC: Splicing up your survival. Nat. Rev. Cancer 15, 574–575 (2015). 10.1038/nrc4013 26383137

[17] Z. E. Stine, C. V. Dang, Splicing and dicing MYC-mediated synthetic lethality. Cancer Cell 28, 405–406 (2015). 26461086 10.1016/j.ccell.2015.09.016

[18] O. Anczukow, A. R. Krainer, The spliceosome, a potential Achilles heel of MYC-driven tumors. Genome Med. 7, 107 (2015). 26490253 10.1186/s13073-015-0234-3 PMC4618744

[19] Y. Shi, J. Yuan, V. Rraklli, E. Maxymovitz, M. Cipullo, M. Liu, S. Li, I. Westerlund, O. C. Bedoya-Reina, P. Bullova, J. Rorbach, C. C. Juhlin, A. Stenman, C. Larsson, P. Kogner, M. J. O’Sullivan, S. Schlisio, J. Holmberg, Aberrant splicing in neuroblastoma generates RNA-fusion transcripts and provides vulnerability to spliceosome inhibitors. Nucleic Acids Res. 49, 2509–2521 (2021). 33555349 10.1093/nar/gkab054 PMC7969022

[20] X. Guo, Q. R. Chen, Y. K. Song, J. S. Wei, J. Khan, Exon array analysis reveals neuroblastoma tumors have distinct alternative splicing patterns according to stage and MYCN amplification status. BMC Med. Genomics 4, 35 (2011). 21501490 10.1186/1755-8794-4-35 PMC3096898

[21] J. Chen, C. S. Hackett, S. Zhang, Y. K. Song, R. J. A. Bell, A. M. Molinaro, D. A. Quigley, A. Balmain, J. S. Song, J. F. Costello, W. C. Gustafson, T. Van Dyke, P.-Y. Kwok, J. Khan, W. A. Weiss, The genetics of splicing in neuroblastoma. Cancer Discov. 5, 380–395 (2015). 25637275 10.1158/2159-8290.CD-14-0892 PMC4390477

[22] S. Zhang, J. S. Wei, S. Q. Li, T. C. Badgett, Y. K. Song, S. Agarwal, C. Coarfa, C. Tolman, L. Hurd, H. Liao, J. He, X. Wen, Z. Liu, C. J. Thiele, F. Westermann, S. Asgharzadeh, R. C. Seeger, J. M. Maris, J. M. G. Auvil, M. A. Smith, E. D. Kolaczyk, J. Shohet, J. Khan, MYCN controls an alternative RNA splicing program in high-risk metastatic neuroblastoma. Cancer Lett. 371, 214–224 (2016). 26683771 10.1016/j.canlet.2015.11.045 PMC4738031

[23] T. Han, M. Goralski, N. Gaskill, E. Capota, J. Kim, T. C. Ting, Y. Xie, N. S. Williams, D. Nijhawan, Anticancer sulfonamides target splicing by inducing RBM39 degradation via recruitment to DCAF15. Science 356, eaal3755 (2017). 28302793 10.1126/science.aal3755

[24] F. M. Behan, F. Iorio, G. Picco, E. Gonçalves, C. M. Beaver, G. Migliardi, R. Santos, Y. Rao, F. Sassi, M. Pinnelli, R. Ansari, S. Harper, D. A. Jackson, R. M. Rae, R. Pooley, P. Wilkinson, D. van der Meer, D. Dow, C. Buser-Doepner, A. Bertotti, L. Trusolino, E. A. Stronach, J. Saez-Rodriguez, K. Yusa, M. J. Garnett, Prioritization of cancer therapeutic targets using CRISPR-Cas9 screens. Nature 568, 511–516 (2019). 30971826 10.1038/s41586-019-1103-9

[25] X. Du, O. A. Volkov, R. M. Czerwinski, H. L. Tan, C. Huerta, E. R. Morton, J. P. Rizzi, P. M. Wehn, R. Xu, D. Nijhawan, E. M. Wallace, Structural basis and kinetic pathway of RBM39 recruitment to DCAF15 by a sulfonamide molecular glue E7820. Structure 27, 1625–1633.e3 (2019). 31693911 10.1016/j.str.2019.10.005

[26] T. B. Faust, H. Yoon, R. P. Nowak, K. A. Donovan, Z. Li, Q. Cai, N. A. Eleuteri, T. Zhang, N. S. Gray, E. S. Fischer, Structural complementarity facilitates E7820-mediated degradation of RBM39 by DCAF15. Nat. Chem. Biol. 16, 7–14 (2020). 31686031 10.1038/s41589-019-0378-3 PMC6917914

[27] D. E. Bussiere, L. Xie, H. Srinivas, W. Shu, A. Burke, C. Be, J. Zhao, A. Godbole, D. King, R. G. Karki, V. Hornak, F. Xu, J. Cobb, N. Carte, A. O. Frank, A. Frommlet, P. Graff, M. Knapp, A. Fazal, B. Okram, S. Jiang, P.-Y. Michellys, R. Beckwith, H. Voshol, C. Wiesmann, J. M. Solomon, J. Paulk, Structural basis of indisulam-mediated RBM39 recruitment to DCAF15 E3 ligase complex. Nat. Chem. Biol. 16, 15–23 (2020). 31819272 10.1038/s41589-019-0411-6

[28] T. Uehara, Y. Minoshima, K. Sagane, N. H. Sugi, K. O. Mitsuhashi, N. Yamamoto, H. Kamiyama, K. Takahashi, Y. Kotake, M. Uesugi, A. Yokoi, A. Inoue, T. Yoshida, M. Mabuchi, A. Tanaka, T. Owa, Selective degradation of splicing factor CAPERα by anticancer sulfonamides. Nat. Chem. Biol. 13, 675–680 (2017). 28437394 10.1038/nchembio.2363

[29] C. Wang, B. Gong, P. R. Bushel, J. Thierry-Mieg, D. Thierry-Mieg, J. Xu, H. Fang, H. Hong, J. Shen, Z. Su, J. Meehan, X. Li, L. Yang, H. Li, P. P. Łabaj, D. P. Kreil, D. Megherbi, S. Gaj, F. Caiment, J. van Delft, J. Kleinjans, A. Scherer, V. Devanarayan, J. Wang, Y. Yang, H.-R. Qian, L. J. Lancashire, M. Bessarabova, Y. Nikolsky, C. Furlanello, M. Chierici, D. Albanese, G. Jurman, S. Riccadonna, M. Filosi, R. Visintainer, K. K. Zhang, J. Li, J.-H. Hsieh, D. L. Svoboda, J. C. Fuscoe, Y. Deng, L. Shi, R. S. Paules, S. S. Auerbach, W. Tong, The concordance between RNA-seq and microarray data depends on chemical treatment and transcript abundance. Nat. Biotechnol. 32, 926–932 (2014). 25150839 10.1038/nbt.3001 PMC4243706

[30] J. M. M. Farland, Z. V. Ho, G. Kugener, J. M. Dempster, P. G. Montgomery, J. G. Bryan, J. M. Krill-Burger, T. M. Green, F. Vazquez, J. S. Boehm, T. R. Golub, W. C. Hahn, D. E. Root, A. Tsherniak, Improved estimation of cancer dependencies from large-scale RNAi screens using model-based normalization and data integration. Nat. Commun. 9, 4610 (2018). 30389920 10.1038/s41467-018-06916-5 PMC6214982

[31] S. Tsubota, S. Kishida, T. Shimamura, M. Ohira, S. Yamashita, D. Cao, S. Kiyonari, T. Ushijima, K. Kadomatsu, PRC2-mediated transcriptomic alterations at the embryonic stage govern tumorigenesis and clinical outcome in MYCN-driven neuroblastoma. Cancer Res. 77, 5259–5271 (2017). 28807939 10.1158/0008-5472.CAN-16-3144

[32] D. Corvetta, O. Chayka, S. Gherardi, C. W. D’Acunto, S. Cantilena, E. Valli, I. Piotrowska, G. Perini, A. Sala, Physical interaction between MYCN oncogene and polycomb repressive complex 2 (PRC2) in neuroblastoma: Functional and therapeutic implications. J. Biol. Chem. 288, 8332–8341 (2013). 23362253 10.1074/jbc.M113.454280 PMC3605651

[33] C. Mc Leod, A. M. Gout, X. Zhou, A. Thrasher, D. Rahbarinia, S. W. Brady, M. Macias, K. Birch, D. Finkelstein, J. Sunny, R. Mudunuri, B. A. Orr, M. Treadway, B. Davidson, T. K. Ard, A. Chiao, A. Swistak, S. Wiggins, S. Foy, J. Wang, E. Sioson, S. Wang, J. R. Michael, Y. Liu, X. Ma, A. Patel, M. N. Edmonson, M. R. Wilkinson, A. M. Frantz, T.-C. Chang, L. Tian, S. Lei, S. M. A. Islam, C. Meyer, N. Thangaraj, P. Tater, V. Kandali, S. Ma, T. Nguyen, O. Serang, I. M. Guire, N. Robison, D. Gentry, X. Tang, L. E. Palmer, G. Wu, E. Suh, L. Tanner, J. M. Murry, M. Lear, A. S. Pappo, Z. Wang, C. L. Wilson, Y. Cheng, S. Meshinchi, L. B. Alexandrov, M. J. Weiss, G. T. Armstrong, L. L. Robison, Y. Yasui, K. E. Nichols, D. W. Ellison, C. Bangur, C. G. Mullighan, S. J. Baker, M. A. Dyer, G. Miller, S. Newman, M. Rusch, R. Daly, K. Perry, J. R. Downing, J. Zhang, St. Jude Cloud—A pediatric cancer genomic data sharing ecosystem. Cancer Discov. 11, 1082–1099 (2021). 33408242 10.1158/2159-8290.CD-20-1230 PMC8102307

[34] T. A. Thanaraj, F. Clark, J. Muilu, Conservation of human alternative splice events in mouse. Nucleic Acids Res. 31, 2544–2552 (2003). 12736303 10.1093/nar/gkg355 PMC156037

[35] T. Berry, W. Luther, N. Bhatnagar, Y. Jamin, E. Poon, T. Sanda, D. Pei, B. Sharma, W. R. Vetharoy, A. Hallsworth, Z. Ahmad, K. Barker, L. Moreau, H. Webber, W. Wang, Q. Liu, A. Perez-Atayde, S. Rodig, N.-K. Cheung, F. Raynaud, B. Hallberg, S. P. Robinson, N. S. Gray, A. D. J. Pearson, S. A. Eccles, L. Chesler, R. E. George, The ALK(F1174L) mutation potentiates the oncogenic activity of MYCN in neuroblastoma. Cancer Cell 22, 117–130 (2012). 22789543 10.1016/j.ccr.2012.06.001 PMC3417812

[36] M. Baur, M. Gneist, T. Owa, C. Dittrich, Clinical complete long-term remission of a patient with metastatic malignant melanoma under therapy with indisulam (E7070). Melanoma Res. 17, 329–331 (2007). 17885589 10.1097/CMR.0b013e3282ef4189

[37] C. Dittrich, A. S. Zandvliet, M. Gneist, A. D. R. Huitema, A. A. J. King, J. Wanders, A phase I and pharmacokinetic study of indisulam in combination with carboplatin. Br. J. Cancer 96, 559–566 (2007). 17285128 10.1038/sj.bjc.6603606 PMC2360043

[38] E. Wang, I. Aifantis, RNA splicing and cancer. Trends Cancer 6, 631–644 (2020). 32434734 10.1016/j.trecan.2020.04.011

[39] S. C. Bonnal, I. Lopez-Oreja, J. Valcarcel, Roles and mechanisms of alternative splicing in cancer—Implications for care. Nat. Rev. Clin. Oncol. 17, 457–474 (2020). 32303702 10.1038/s41571-020-0350-x

[40] C. L. Hirsch, Z. C. Akdemir, L. Wang, G. Jayakumaran, D. Trcka, A. Weiss, J. J. Hernandez, Q. Pan, H. Han, X. Xu, Z. Xia, A. P. Salinger, M. Wilson, F. Vizeacoumar, A. Datti, W. Li, A. J. Cooney, M. C. Barton, B. J. Blencowe, J. L. Wrana, S. Y. R. Dent, Myc and SAGA rewire an alternative splicing network during early somatic cell reprogramming. Genes Dev. 29, 803–816 (2015). 25877919 10.1101/gad.255109.114 PMC4403257

[41] A. A. Hoskins, L. J. Friedman, S. S. Gallagher, D. J. Crawford, E. G. Anderson, R. Wombacher, N. Ramirez, V. W. Cornish, J. Gelles, M. J. Moore, Ordered and dynamic assembly of single spliceosomes. Science 331, 1289–1295 (2011). 21393538 10.1126/science.1198830 PMC3086749

[42] W. Chen, M. J. Moore, Spliceosomes. Curr. Biol. 25, R181–R183 (2015). 25734262 10.1016/j.cub.2014.11.059

[43] G. A. Stepanyuk, P. Serrano, E. Peralta, C. L. Farr, H. L. Axelrod, M. Geralt, D. Das, H.-J. Chiu, L. Jaroszewski, A. M. Deacon, S. A. Lesley, M.-A. Elsliger, A. Godzik, I. A. Wilson, K. Wüthrich, D. R. Salomon, J. R. Williamson, UHM-ULM interactions in the RBM39-U2AF65 splicing-factor complex. Acta Crystallogr. D Struct. Biol. 72( Pt. 4), 497–511 (2016). 27050129 10.1107/S2059798316001248 PMC4822562

[44] M. Tari, V. Manceau, J. de Matha Salone, A. Kobayashi, D. Pastré, A. Maucuer, U2AF65 assemblies drive sequence-specific splice site recognition. EMBO Rep. 20, e47604 (2019). 31271494 10.15252/embr.201847604 PMC6681011

[45] M. Schapira, M. F. Calabrese, A. N. Bullock, C. M. Crews, Targeted protein degradation: Expanding the toolbox. Nat. Rev. Drug Discov. 18, 949–963 (2019). 31666732 10.1038/s41573-019-0047-y

[46] M. Jung, A. J. Russell, C. Kennedy, A. J. Gifford; Australian Ovarian Cancer Study Group, K.-A. Mallitt, S. Sivarajasingam, D. D. Bowtell, A. De Fazio, M. Haber, M. D. Norris, M. J. Henderson, Clinical importance of Myc family oncogene aberrations in epithelial ovarian cancer. JNCI Cancer Spectr. 2, pky047 (2018). 31360864 10.1093/jncics/pky047 PMC6649713

[47] A. Dobin, C. A. Davis, F. Schlesinger, J. Drenkow, C. Zaleski, S. Jha, P. Batut, M. Chaisson, T. R. Gingeras, STAR: Ultrafast universal RNA-seq aligner. Bioinformatics 29, 15–21 (2013). 23104886 10.1093/bioinformatics/bts635 PMC3530905

[48] J. D. Buenrostro, P. G. Giresi, L. C. Zaba, H. Y. Chang, W. J. Greenleaf, Transposition of native chromatin for fast and sensitive epigenomic profiling of open chromatin, DNA-binding proteins and nucleosome position. Nat. Methods 10, 1213–1218 (2013). 24097267 10.1038/nmeth.2688 PMC3959825

[49] J. D. Buenrostro, B. Wu, H. Y. Chang, W. J. Greenleaf, ATAC-seq: A method for assaying chromatin accessibility genome-wide. Curr. Protoc. Mol. Biol. 109, 21.29.1–21.29.9 (2015). 10.1002/0471142727.mb2129s109 PMC4374986 25559105

[50] H. Li, R. Durbin, Fast and accurate long-read alignment with Burrows-Wheeler transform. Bioinformatics 26, 589–595 (2010). 20080505 10.1093/bioinformatics/btp698 PMC2828108

[51] H. Li, B. Handsaker, A. Wysoker, T. Fennell, J. Ruan, N. Homer, G. Marth, G. Abecasis, R. Durbin; Genome Project Data Processing Subgroup, The Sequence Alignment/Map format and SAMtools. Bioinformatics 25, 2078–2079 (2009). 19505943 10.1093/bioinformatics/btp352 PMC2723002

[52] Y. Zhang, T. Liu, C. A. Meyer, J. Eeckhoute, D. S. Johnson, B. E. Bernstein, C. Nusbaum, R. M. Myers, M. Brown, W. Li, X. S. Liu, Model-based analysis of ChIP-Seq (MACS). Genome Biol. 9, R137 (2008). 18798982 10.1186/gb-2008-9-9-r137 PMC2592715

[53] A. R. Quinlan, I. M. Hall, BEDTools: A flexible suite of utilities for comparing genomic features. Bioinformatics 26, 841–842 (2010). 20110278 10.1093/bioinformatics/btq033 PMC2832824

[54] S. Heinz, C. Benner, N. Spann, E. Bertolino, Y. C. Lin, P. Laslo, J. X. Cheng, C. Murre, H. Singh, C. K. Glass, Simple combinations of lineage-determining transcription factors prime cis-regulatory elements required for macrophage and B cell identities. Mol. Cell 38, 576–589 (2010). 20513432 10.1016/j.molcel.2010.05.004 PMC2898526

[55] H. Thorvaldsdottir, J. T. Robinson, J. P. Mesirov, Integrative Genomics Viewer (IGV): High-performance genomics data visualization and exploration. Brief. Bioinform. 14, 178–192 (2013). 22517427 10.1093/bib/bbs017 PMC3603213

[56] A. Fix, C. Lucchesi, A. Ribeiro, D. Lequin, G. Pierron, G. Schleiermacher, O. Delattre, I. Janoueix-Lerosey, Characterization of amplicons in neuroblastoma: High-resolution mapping using DNA microarrays, relationship with outcome, and identification of overexpressed genes. Genes Chromosomes Cancer 47, 819–834 (2008). 18553563 10.1002/gcc.20583

[57] P. Shannon, A. Markiel, O. Ozier, N. S. Baliga, J. T. Wang, D. Ramage, N. Amin, B. Schwikowski, T. Ideker, Cytoscape: A software environment for integrated models of biomolecular interaction networks. Genome Res. 13, 2498–2504 (2003). 14597658 10.1101/gr.1239303 PMC403769

[58] S. Maere, K. Heymans, M. Kuiper, BiNGO: A Cytoscape plugin to assess overrepresentation of gene ontology categories in biological networks. Bioinformatics 21, 3448–3449 (2005). 15972284 10.1093/bioinformatics/bti551

[59] L.-A. Van de Velde, E. K. Allen, J. C. Crawford, T. L. Wilson, C. S. Guy, M. Russier, L. Zeitler, A. Bahrami, D. Finkelstein, S. Pelletier, S. Schultz-Cherry, P. G. Thomas, P. J. Murray, Neuroblastoma formation requires unconventional CD4 T cells and Arginase-1-dependent myeloid cells. Cancer Res. 81, 5047–5059 (2021). 34301764 10.1158/0008-5472.CAN-21-0691 PMC8488023

[60] M. G. Rees, B. Seashore-Ludlow, J. H. Cheah, D. J. Adams, E. V. Price, S. Gill, S. Javaid, M. E. Coletti, V. L. Jones, N. E. Bodycombe, C. K. Soule, B. Alexander, A. Li, P. Montgomery, J. D. Kotz, C. S.-Y. Hon, B. Munoz, T. Liefeld, V. Dančík, D. A. Haber, C. B. Clish, J. A. Bittker, M. Palmer, B. K. Wagner, P. A. Clemons, A. F. Shamji, S. L. Schreiber, Correlating chemical sensitivity and basal gene expression reveals mechanism of action. Nat. Chem. Biol. 12, 109–116 (2016). 26656090 10.1038/nchembio.1986 PMC4718762

