# Supplementary material for: Targeting the spliceosome through RBM39 degradation results in exceptional responses in high-risk neuroblastoma models
Source: Sci Adv. 2021 Nov 17;7(47):eabj5405. doi: 10.1126/sciadv.abj5405 (PMC8598007; doi:10.1126/sciadv.abj5405)
Supplement: Supplementary file 1 — Figs S1 to S13 Legends for tables S1 to S12 [file sciadv.abj5405_sm.pdf]

Science Advances
AAAS

Supplementary Materials for

Targeting the spliceosome through RBM39 degradation results in exceptional responses in high-risk neuroblastoma models

Shivendra Singh, Waise Quarni, Maria Goralski, Shibiao Wan, Hongjian Jin, Lee-Ann Van de Velde, Jie Fang, Qiong Wu, Ahmed Abu-Zaid, Tingting Wang, Ravi Singh, David Craft, Yiping Fan, Thomas Confer, Melissa Johnson, Walter J. Akers, Ruoning Wang, Peter J. Murray, Paul G. Thomas, Deepak Nijhawan*, Andrew M. Davidoff1*, Jun Yang*

*Corresponding author. Email: jun.yang2@stjude.org (J.Y.); deepak.nijhawan@utsouthwestern.edu (D.N.); andrew.davidoff@stjude.org (A.M.D.)

Published 17 November 2021, *Sci. Adv.* **7**, eabj5405 (2021)
DOI: 10.1126/sciadv.abj5405

The PDF file includes:

Figs S1 to S13
Legends for tables S1 to S12

Other Supplementary Material for this manuscript includes the following:

Tables S1 to S12

SUPPLEMENTAL FIGURES AND LEGENDS

A

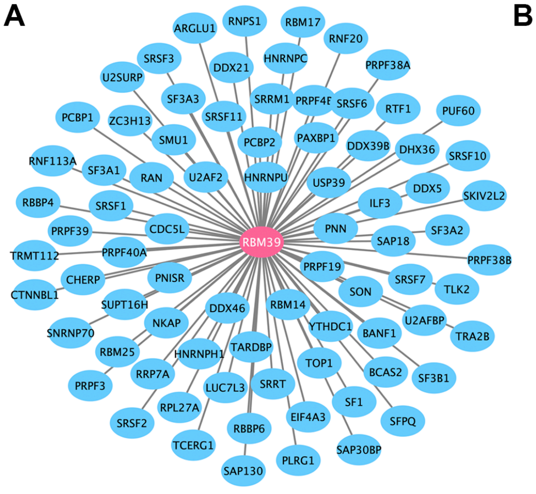

B

Gene set enrichment analysis of RBM39 interactome

Gene Set Name	p-value	FDR q-value
FISCHER_DREAM_TARGETS	1.58E-16	7.58E-14
HAMAI_APOPTOSIS_VIA_TRAIL_UP	1.69E-13	6.31E-11
BIDUS_METASTASIS_UP	6.49E-13	1.82E-10
BILD_CTNNB1_ONCOGENIC_SIGNATURE	2.00E-10	3.74E-08
WANG_TUMOR_INVASIVENESS_UP	2.50E-10	4.22E-08
MARKEY_RB1_ACUTE_LOF_UP	9.99E-10	1.40E-07
BENPORATH_CYCLING_GENES	5.17E-09	5.44E-07
PAL_PRMT5_TARGETS_UP	6.92E-09	7.00E-07
DANG_BOUND_BY_MYC	1.78E-08	1.58E-06
BENPORATH_NANOG_TARGETS	6.31E-08	4.62E-06

C D E

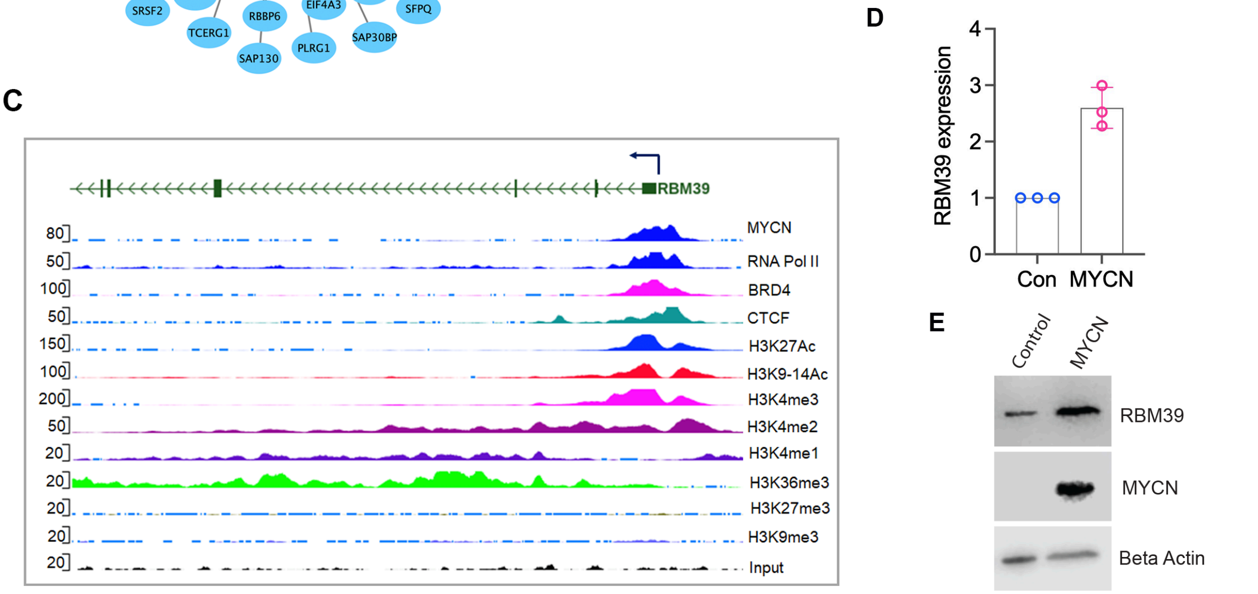

Supplemental Figure 1. RBM39 subnetwork and regulation by MYC. A. The 1585 essential fitness genes to neuroblastoma cell lines (n=7, dependency score>40%) identified through genome-wide CRISPR/Cas9 library screen by Sanger institute were uploaded into STRING program (https://string-db.org) for network interaction analysis with confidence threshold 0.4. The RBM39 subnetwork showing direct interaction with RBM39 is uploaded into Cytoscape program for presentation. **B**. Gene set enrichment analysis of RBM39 interactome using GCP database (Genetic and Chemical Perturbation) showing the pathway and function enrichment. **C**. The epigenetic landscape of RBM39 genomic locus showing the MYCN binding, in line with the binding of BRD4, CTCF and RNA polymerase II, and active transcriptional marks of histone modifications. **D**. Real-time PCR showing that *RBM39* mRNA is induced by MYCN overexpressed in SK-N-SH cells. **E**. Western blot showing that RBM39 at protein levels is induced by MYCN overexpressed in SK-N-SH cells.

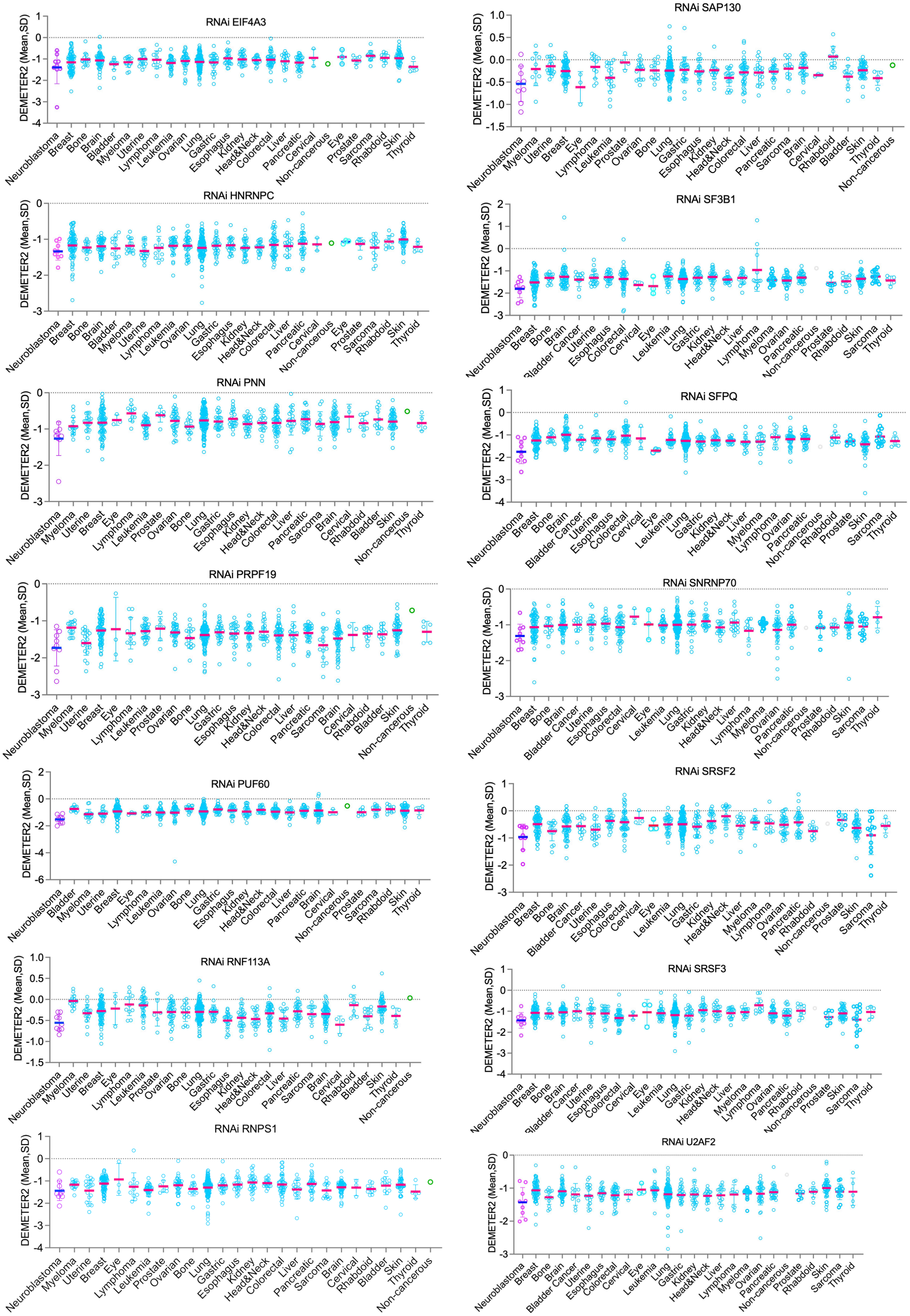
RNAi EIF4A3
RNAi SAP130
RNAi HNRNPC
RNAi SF3B1
RNAi PNN
RNAi SFPQ
RNAi PRPF19
RNAi SNRNP70
RNAi PUF60
RNAi SRSF2
RNAi RNF113A
RNAi SRSF3
RNAi RNPS1
RNAi U2AF2
DEMETER2 (Mean,SD)

Supplemental Figure 2. Dependency of neuroblastoma on splicing factors with direct interaction with RBM39. The DEMETER2 score of an RNAi screen across 25 cancer lineages showing neuroblastoma has a greater survival dependency on RBM39 interacting splicing factors than other cancer lineages. A lower DEMETER2 score indicates a higher likelihood that a gene of interest is essential in a given cell line. The DETERM2 score was represented by mean ±SD.

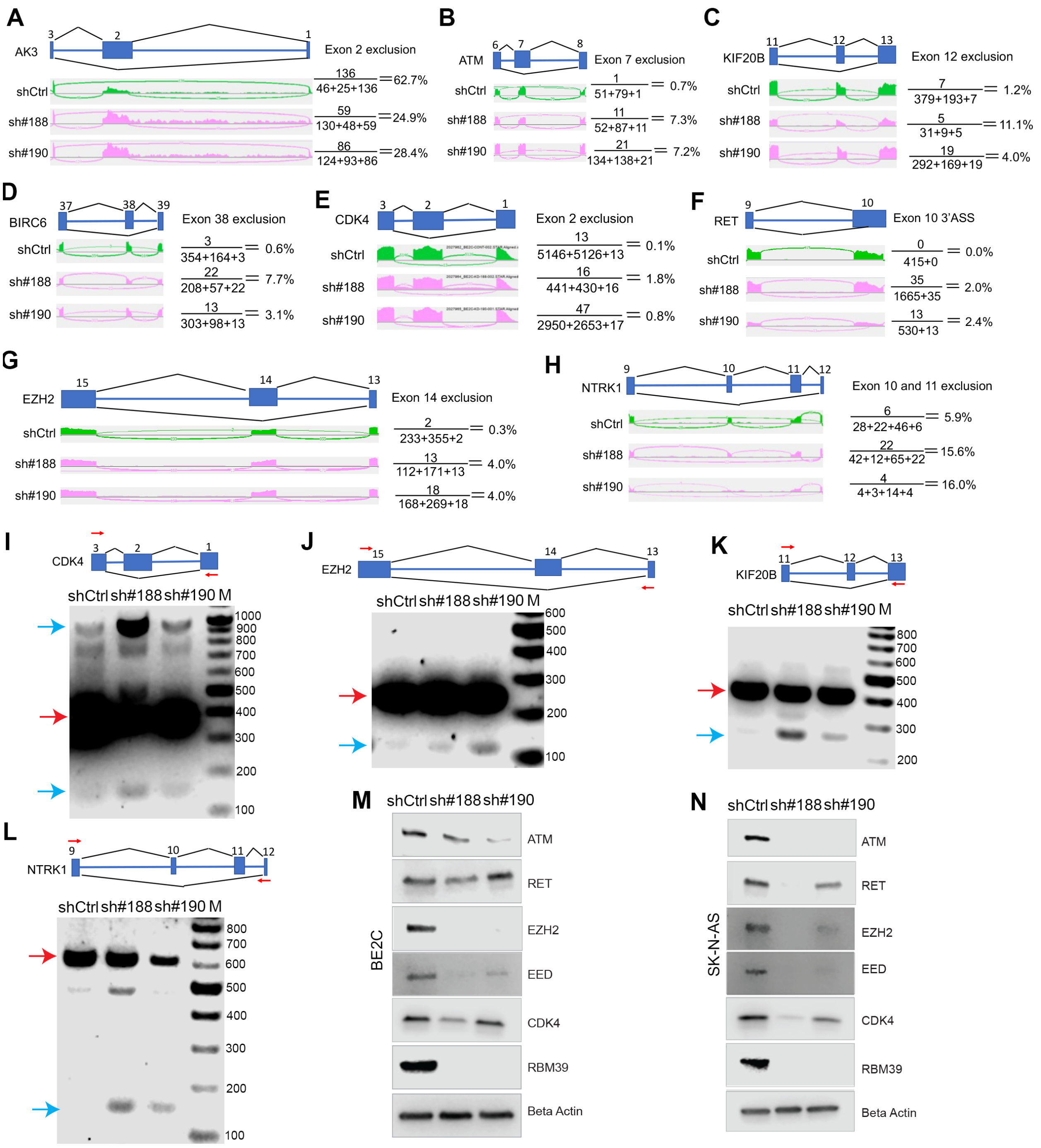

Supplemental Figure 3. RBM39 regulates alternative splicing of genes. A-H. Sashimi plots using IGV program showing the splicing changes of selected genes in BE2C cells after RBM39 knockdown with two different shRNAs (sh#188, sh#190), which indicates the mis-splicing by loss of function of RBM39. The numbers indicate the read counts of exon-intron junctions of RNA-seq. The equation shows the percentage of exon usage after RBM39 depletion. **I-L**. Reverse transcription PCR validating the mis-splicing events induced by RBM39 knockdown in BE2C cells for 72h. PCR products loaded into 1-2% agarose gel with ethidium bromide for electrophoresis. Red color indicates the predicted molecular weight of PCR products. Blue color indicates the mis-spliced PCR products. Note, CDK4 shows one band with molecular weight of 1000nt in length, which is not the predicted size of spliced exons and may thus indicate intron retention leading to large size of PCR products. PCR primer positions indicated by short red arrows at both ends of exons. **M-N.** Western blot assessment of expression of indicated molecules that undergo aberrant splicing by RBM39 knockdown in BE2C cells and SK-N-AS cells.

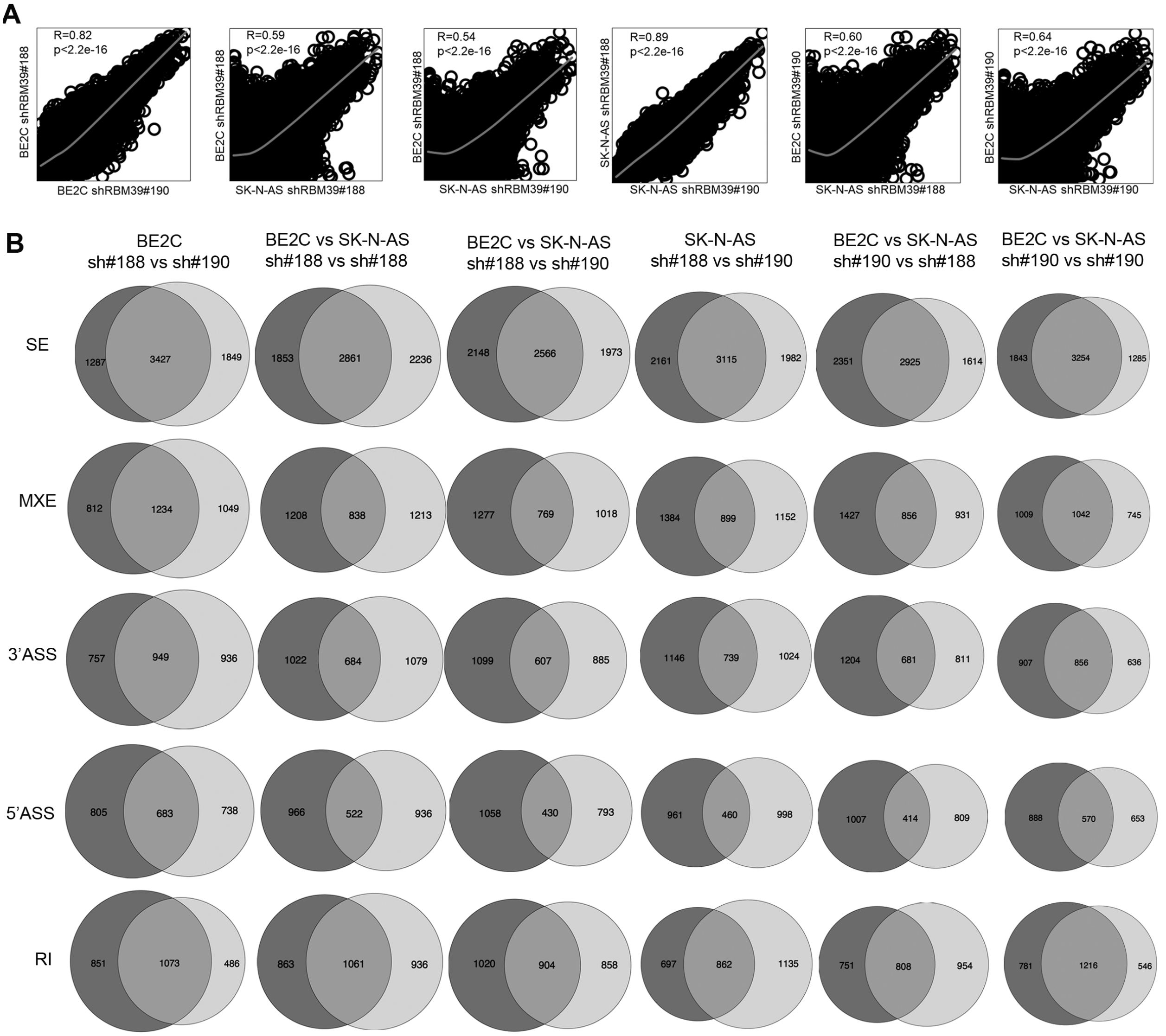

Supplemental Figure 4. Comparison of splicing events induced by RBM39 knockdown in BE2C and SK-N-AS cells. (A) The genome-wide pairwise correlation of splicing changes by RBM39 knockdown with 2 different shRNAs is performed to compare the consistency between shRNAs and cell lines. (**B**) Venn diagram showing the common splicing events (FDR < 0.05 & abs(IncLevelDifference)>0.1) induced by RBM39 knockdown (sh#188, sh#190) in BE2C and SK-N-AS cells. A3SS= alternative 3’ splicing, A5SS= alternative 5’ splicing, MXE= mutually exclusive exon, RI= intron, retention, SE= skipped exon.

A

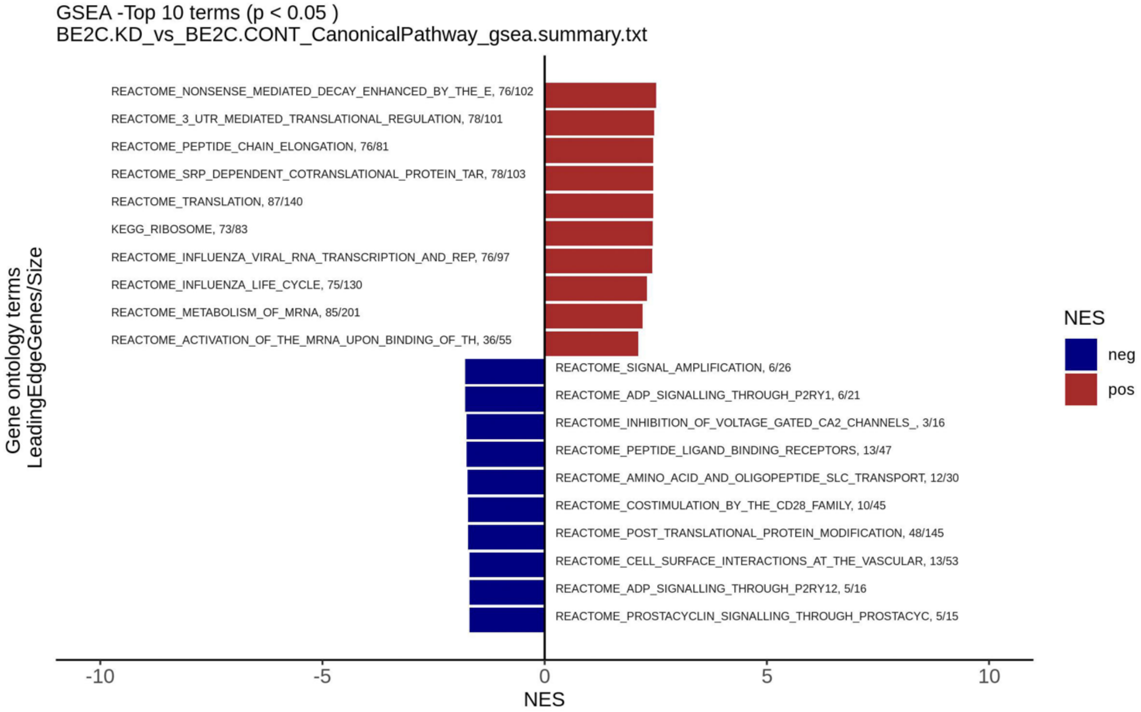

B

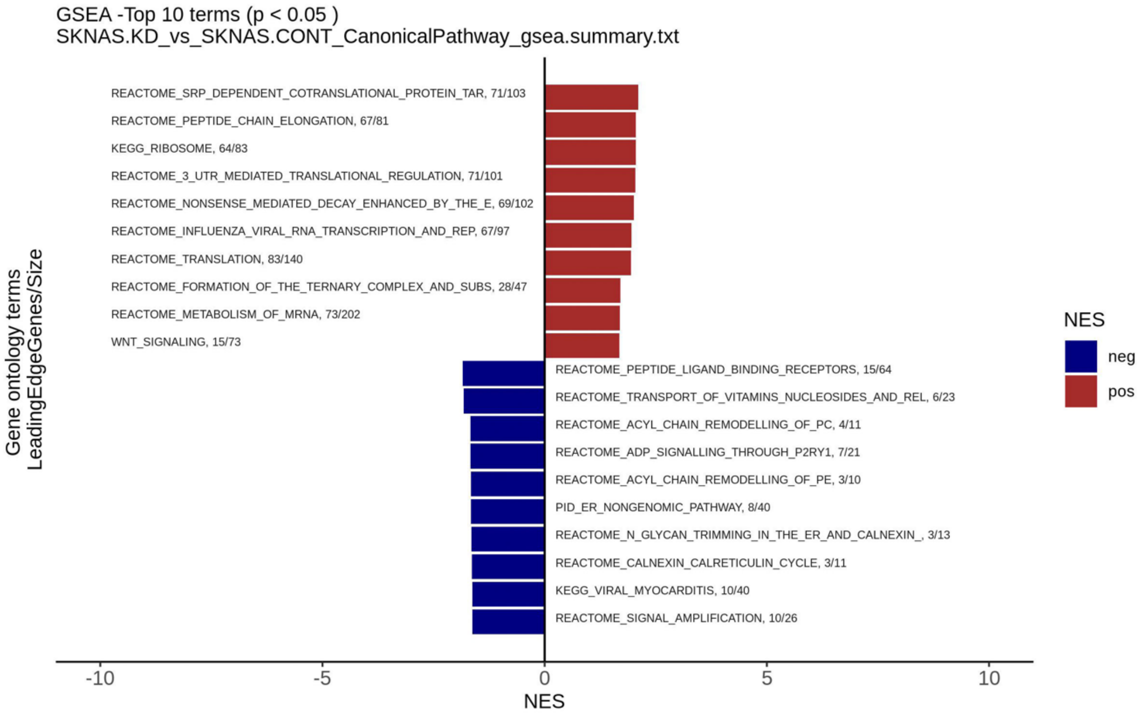

Supplemental Figure 5. GSEA analysis of altered pathways after RBM39 knockdown in BE2C and SK-N-AS cells.

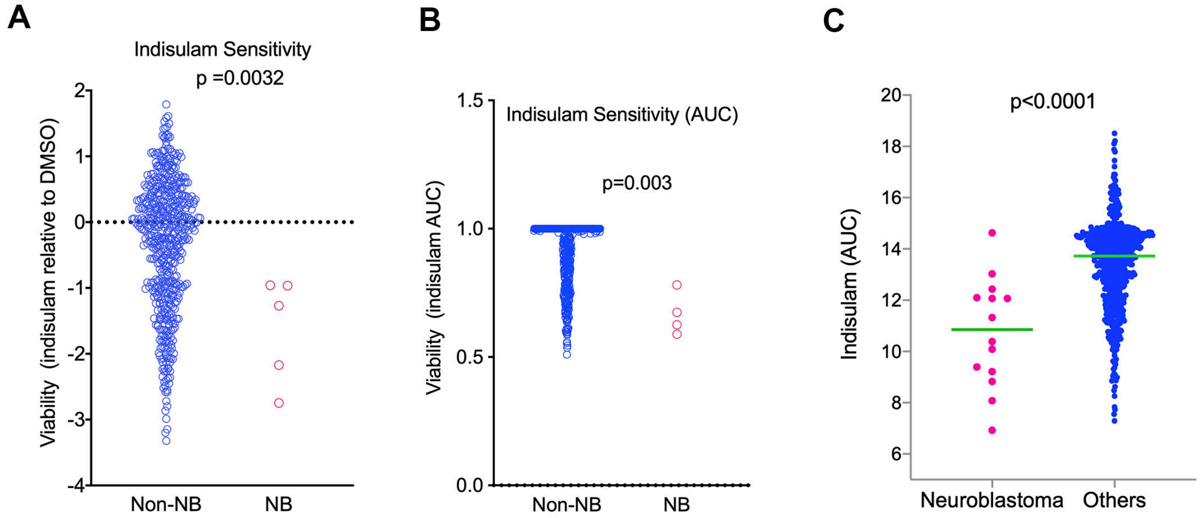

Supplemental Figure 6. Neuroblastoma cells are selectively sensitive to indisulam. **A**. PRISM primary screen with single dose of indisulam shows the viability score between neuroblastoma cell lines (NB=5) and other cancer cell lines (non-NB=550). P values were calculated using unpaired student t test. **B**. PRISM secondary screen with area under curve (AUC) of for the potent compounds shows the indisulam selectivity against neuroblastoma cell lines (NB=4) over other cell lines (non-NB=470). P values were calculated using unpaired student t test. **C**. An independent screen from CTRP shows the area under curve (AUC) of indisulam in neuroblastoma cell lines (n=14) and other cancer lineages (n=707). P values were calculated using unpaired student t test.

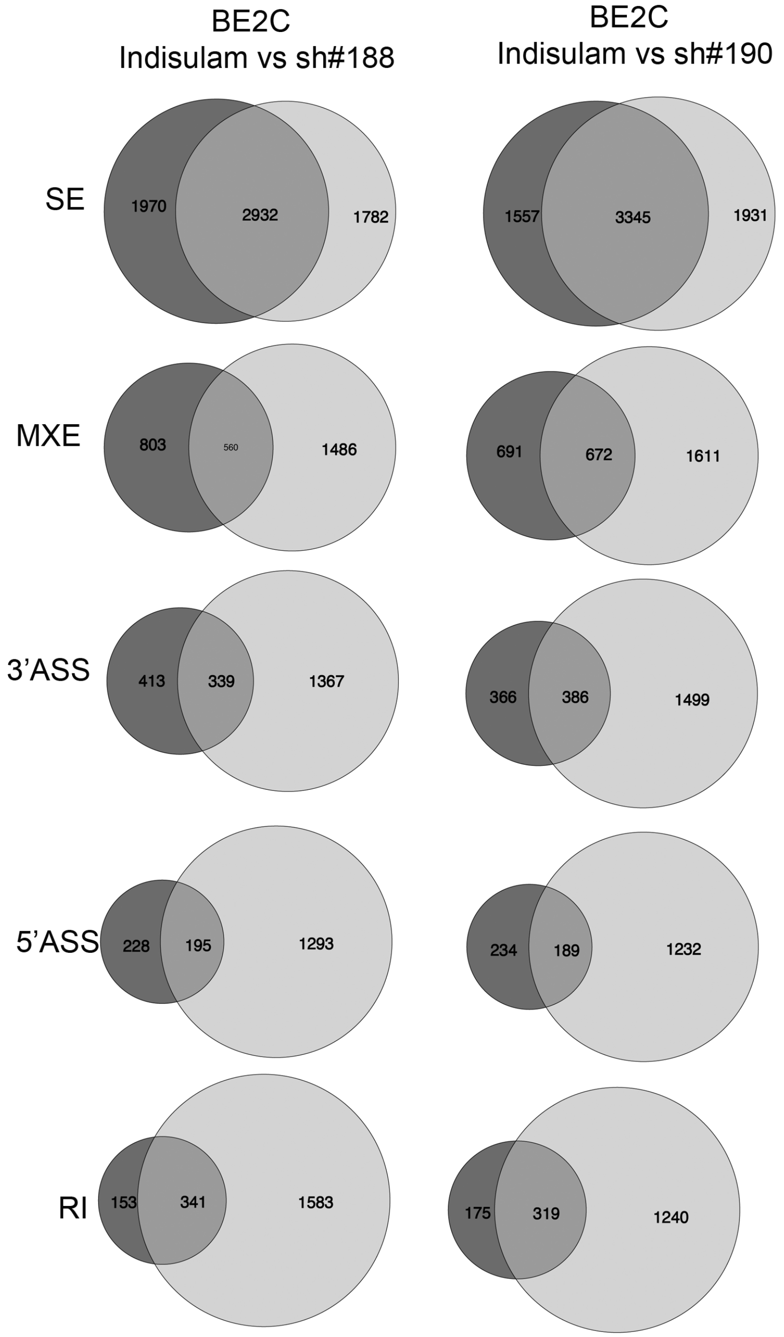

Supplemental Figure 7. Comparison of splicing events induced by indisulam and RBM39 knockdown in BE2C cells. Venn diagram showing the common splicing events (FDR < 0.05 & abs(IncLevelDifference)>0.1) induced by indisulam and RBM39 knockdown (sh#188, sh#190) in BE2C cells. A3SS= alternative 3’ splicing, A5SS= alternative 5’ splicing, MXE= mutually exclusive exon, RI= intron, retention, SE= skipped exon.

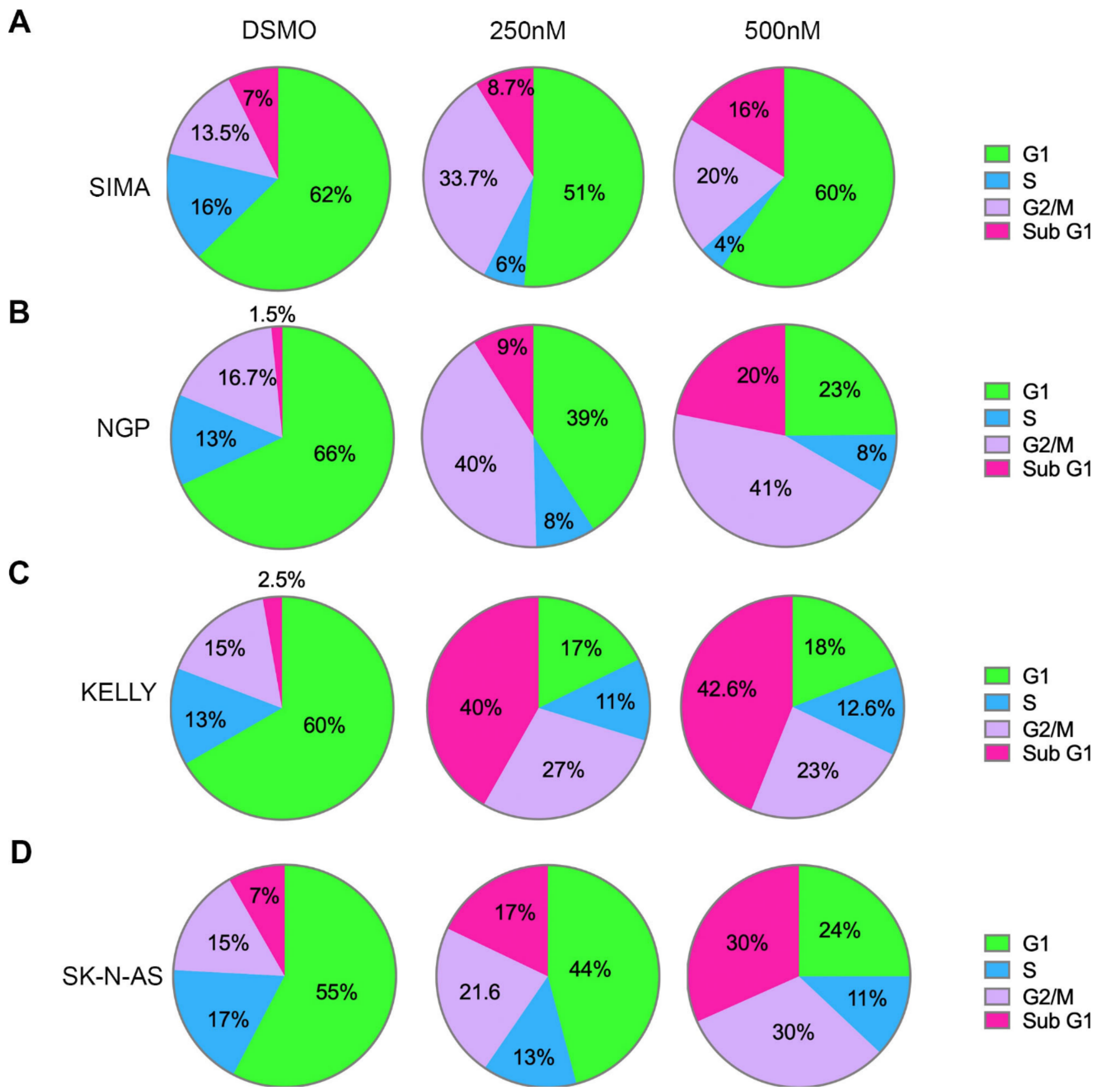

Supplemental Figure 8. Indisulam induces G2/M phase arrest and cell death. Pie chart plots show the percentage of each phase of cell cycle analyzed by FACS after neuroblastoma cells were treated with control, 250nM indisulam and 500 nM indisulam, respectively, for 48 hours.

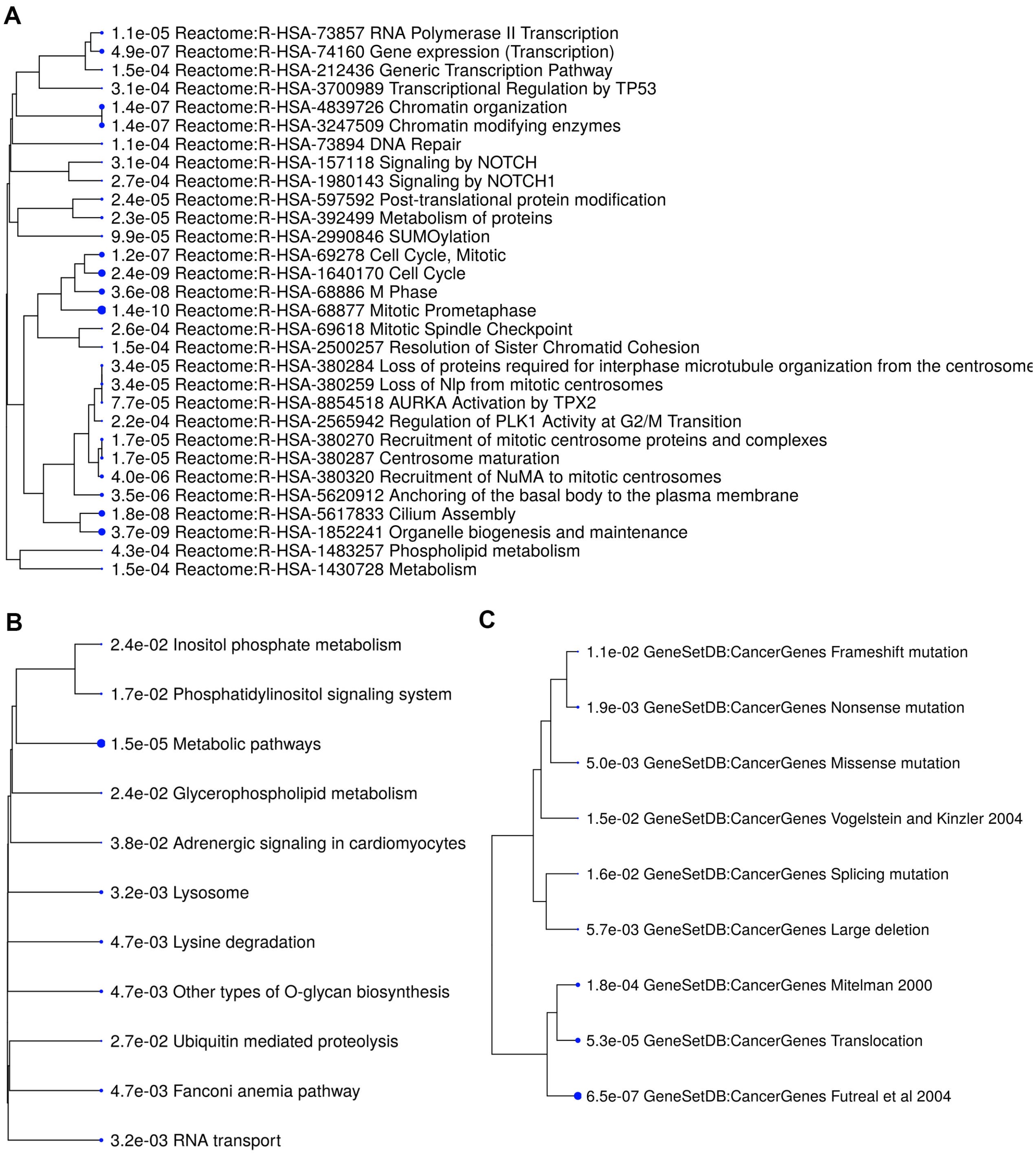

Supplemental Figure 9. Pathway analysis of genes with altered splicing induced by RBM39 knockdown and indisulam treatment in BE2C cells. ShinyGO v0.61: Gene Ontology Enrichment Analysis program is used to analyze pathway enrichment.

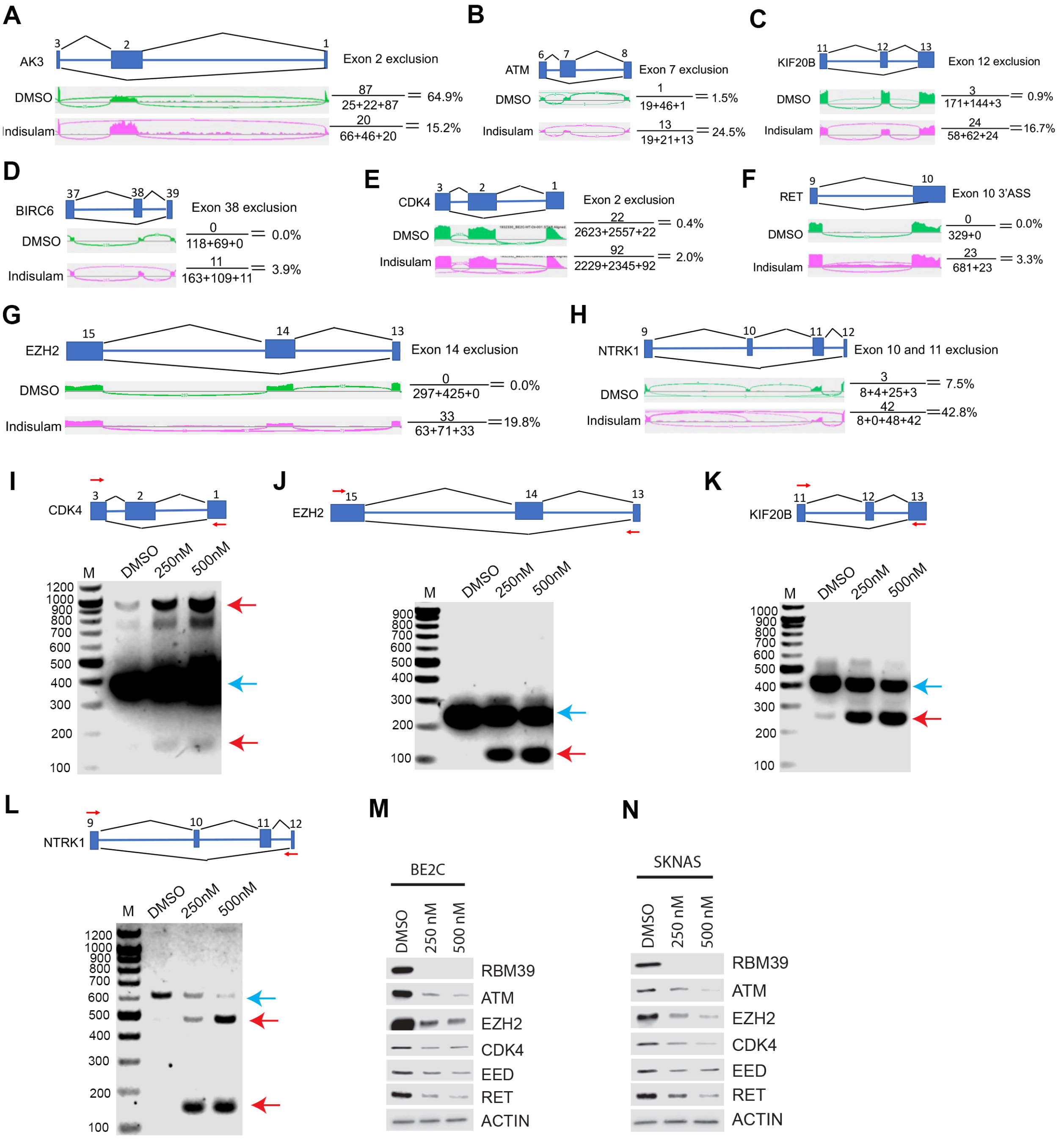

Supplemental Figure 10. Degradation of RBM39 leads to alterations of alternative splicing. A-H. Sashimi plots using IGV program showing the splicing changes of selected genes in BE2C cells after RBM39 degradation in BE2C cells by 250nM and 500nM of indisulam, which indicates the mis-splicing by loss of function of RBM39. The numbers indicate the read counts of exon-intron junctions of RNA-seq. The equation shows the percentage of exon usage after RBM39 degradation. **I-L**. Reverse transcription PCR validating the mis-splicing events induced by RBM39 degradation in BE2C cells for 48h. PCR products loaded into 1-2% agarose gel with ethidium

bromide for electrophoresis. Red color indicates the predicted molecular weight of PCR products. Blue color indicates the mis-spliced PCR products. Note, CDK4 shows one band with molecular weight of 1000nt in length, which is not the predicted size of spliced exons and may indicate intron retention leading to large size of PCR products. NTRK1 also shows one additional spliced isoform at 500nt in length, suggesting only one exon is spliced out. PCR primer positions indicated by short red arrows at both ends of exons. **M-N.** Western blot assessment of expression of indicated molecules that undergo aberrant splicing by RBM39 degradation for 48h in BE2C cells and SK-N-AS cells.

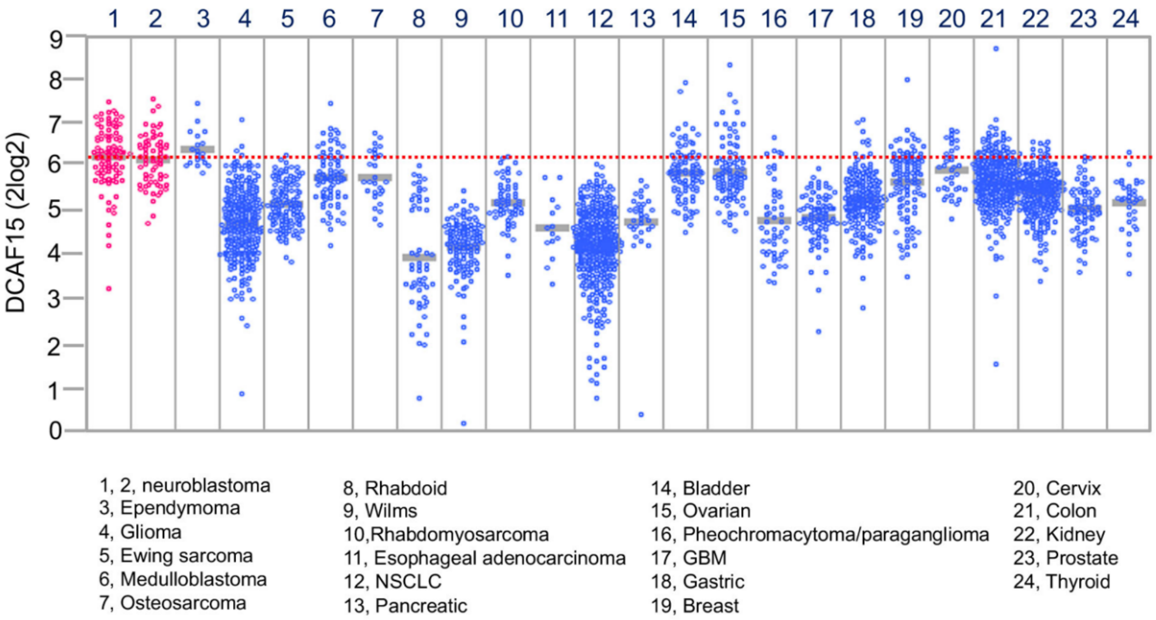

Supplemental Figure 11. RBM39 expression in a variety of cancer datasets. Data were extracted using R2: Genomics Analysis and Visualization Platform.

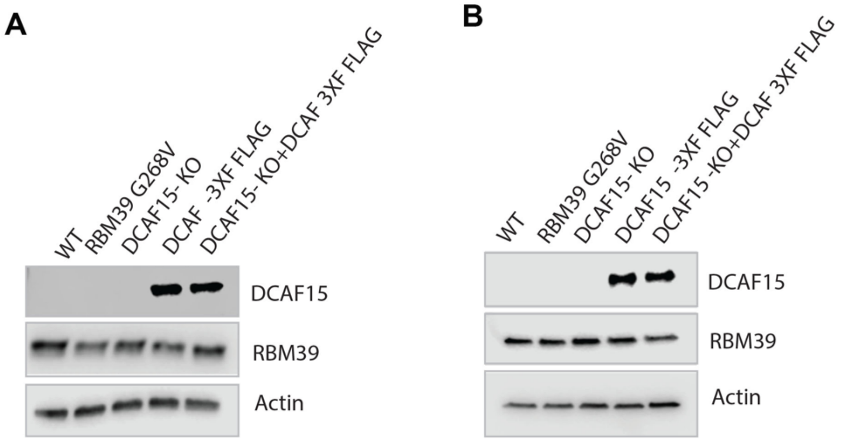

Supplemental Figure 12. Western blot assessement of RBM39 expression in BE2C (A) and SK-N-AS (B) cells and their derivatives that overexpression DCAF15 (DCAF15 3XF), knockout (DCAF15 KO), rescue (DCAF15 KO + DCAF15 3XF), and RBM39 mutant (G268V).

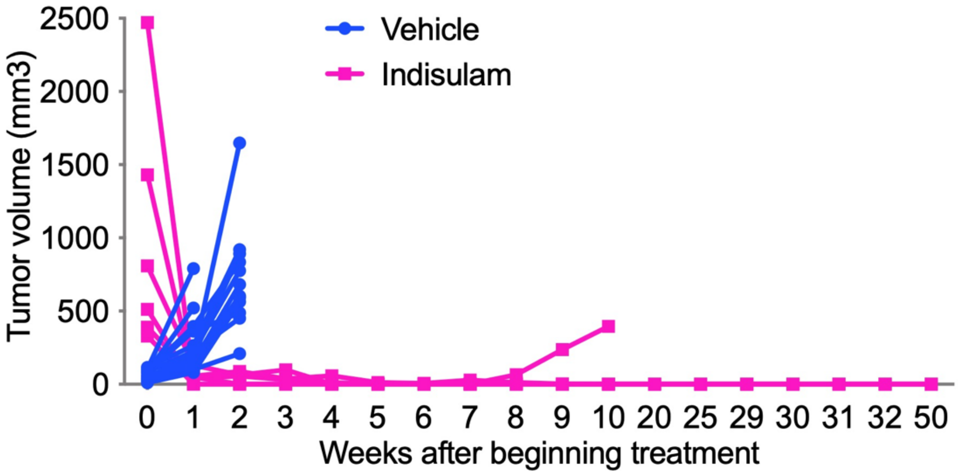

Supplemental Figure 13. Follow up of individual tumor volume for transgenic MYCN/ALK^{F1178L} mice that were treated with vehicle (n=10) and 25mg/kg of indisulam (n=6), standard schedule.

Supplementary Table Legends

Supplementary Table 1: Differentially expressed genes in high-risk vs low-risk neuroblastomas.

Supplementary Table 2: Essential fitness genes in neuroblastoma cells identified by genome-wide CRISPR screen.

Supplementary Table 3: Dependency genes in 707 cancer cell lines by genome-wide shRNA screen.

Supplementary Table 4: Alternative splicing changes induced by RBM39 knockdown in BE2C cells.

Supplementary Table 5: Alternative splicing changes induced by RBM39 knockdown in SK-N-AS cells.

Supplementary Table 6: Cancer genes mis-spliced by RBM39 knockdown.

Supplementary Table 7: Differentially expressed genes induced by RBM39 knockdown.

Supplementary Table 8: Mean log-fold difference between neuroblastoma and non-neuroblastoma cell lines for compounds screened by PRISM.

Supplementary Table 9: Mean AUC difference for compounds screened in neuroblastoma cell lines by PRISM.

Supplementary Table 10: AUC for indisulam screened in cell lines by CTRP.

Supplementary Table 11: *DCAF15* expression in cell lines screened by CTRP.

Supplementary Table 12: *DCAF15* expression pediatric cancers.

.
